# Glymphatic dysfunction in Alzheimer's disease: a systematic review and meta-analysis of the DTI-ALPS index as an imaging biomarker

**DOI:** 10.3389/fnagi.2026.1832525

**Published:** 2026-07-02

**Authors:** Yutong Wu, Chen Zhang, Jiayi Yan, Lan Lin, Yuan Tian, Zhenrong Fu, Xiaojun Sun

**Affiliations:** 1 Key Laboratory of Adolescent CyberPsychology and Behavior (CCNU), Ministry of Education, Wuhan, China; 2 Key Laboratory of Human Development and Mental Health of Hubei Province, School of Psychology, Central China Normal University, Wuhan, China; 3 Department of Medical Engineering, Beijing Hospital of Traditional Chinese Medicine, Capital Medical University, Beijing, China; 4 Department of Biomedical Engineering, College of Chemistry and Life Science, Beijing University of Technology, Beijing, China; 5 Intelligent Physiological Measurement and Clinical Translation, Beijing International Base for Scientific and Technological Cooperation, Beijing University of Technology, Beijing, China

**Keywords:** Alzheimer's disease, biomarker, DTI-ALPS, glymphatic function, neuroimaging

## Abstract

**Introduction:**

Although the diffusion tensor image analysis along the perivascular space (DTI-ALPS) index is widely utilized as a proxy for glymphatic function in Alzheimer's Disease (AD) research, its association with core AD pathological biomarkers remains inconclusive due to inter-study heterogeneity. This systematic review aimed to investigate associations between the DTI-ALPS index and AD biomarkers and to elucidate potential sources of heterogeneity.

**Methods:**

The study protocol adhered to PRISMA guidelines and was registered with PROSPERO (CRD420251266067). We systematically searched Web of Science, PubMed, and ScienceDirect from January 1, 2017 to September 18, 2025 for studies examining the relationship between the DTI-ALPS index and core AD biomarkers. Random-effects meta-analyses were conducted when studies employed comparable methodologies and reported effect sizes while narrative synthesis was performed otherwise. Methodological quality was assessed using the Newcastle–Ottawa Scale (NOS) and the risk of bias was evaluated using the Risk of Bias in Systematic reviews (ROBIS) tool.

**Results:**

Thirty-six studies were included. Meta-analyses revealed significant correlations between the DTI-ALPS index (*N* = 22) and Aβ PET deposition (*N* = 9), MMSE (*N* = 15), and MoCA (*N* = 10) scores. Conversely, the association with tau PET deposition (*N* = 3) was not significant after adjusting for publication bias. Regarding clinical staging, indices were significantly lower in AD and mild cognitive impairment groups compared to controls but no difference was found between the two patient groups. Based on meta-regression and narrative synthesis results, we identified methodological variability and AD pathological complexity as primary sources of heterogeneity.

**Discussion:**

Consequently, we proposed the “GS Hub Integrative Framework.” This framework posits that vascular, genetic, lifestyle, and environmental risk factors converge on glymphatic dysfunction (DTI-ALPS index) as a central hub, accelerating Aβ/tau accumulation, neurodegeneration, and cognitive decline. Future methodological standardization and refined patient stratification are essential for clinical translation.

**Systematic review registration:**

https://www.crd.york.ac.uk/PROSPERO/view/CRD420251266067, identifier: CRD420251266067.

## 1 Introduction

The glymphatic system (GS) serves as a critical macroscopic waste clearance pathway in the central nervous system (Iliff et al., 2012; Supplementary Section 1.1). Its primary function is to remove metabolic byproducts, including β-amyloid (Aβ) and tau, through the perivascular spaces (PVS). Dysfunction of the GS is now recognized as a pivotal driver of pathological protein aggregation and neurodegeneration in Alzheimer's disease (AD; Nedergaard and Goldman, 2020). Under the amyloid/tau/neurodegeneration (A/T/N) framework, the deposition of pathological proteins precedes cognitive decline (Jack et al., 2013, 2016). This sequence underscores the critical role of assessing GS function as a target for early identification and intervention in AD.

However, *in vivo* assessment of GS function faces significant technical challenges. Although intrathecal tracer injection remains the gold standard, its invasiveness limits widespread clinical application (Eide and Ringstad, 2015). To address this, the diffusion tensor image analysis along the perivascular space (DTI-ALPS) has emerged as a leading non-invasive surrogate (Taoka et al., 2017; Supplementary Section 1.2). By quantifying water diffusivity within the PVS, the DTI-ALPS index provides an indirect measure that facilitates large-scale clinical assessment of GS function.

Despite its immense potential, the validity of the DTI-ALPS index as an AD pathological biomarker remains a subject of intense debate. Some studies report that reduced DTI-ALPS index are significantly associated with increased Aβ or tau burden, even in preclinical stages (Hsu et al., 2023; Huang et al., 2024; Li et al., 2024). Conversely, other studies have failed to replicate these associations. More critically, recent evidence suggests that DTI-ALPS measurements may be confounded by non-specific white matter microstructural degeneration (Li et al., 2025). This finding questions its specificity in reflecting glymphatic clearance function. These conflicting findings regarding the relationship between DTI-ALPS and core AD pathology hinder its translation from a research tool to a reliable clinical biomarker.

Previous quantitative syntheses have primarily confirmed the diagnostic efficacy of DTI-ALPS in distinguishing AD patients from controls (Costa et al., 2024). While these studies partially addressed its association with core AD neuropathology (Khalafi et al., 2025), they failed to resolve the heterogeneity of the results. To bridge this gap, this study systematically reviews the relationship between the DTI-ALPS index and AD biomarkers, including Aβ, tau, and neurodegeneration. We identify methodological discrepancies and pathological complexity as the primary sources of conflicting outcomes. On this basis, we propose the “GS Hub Integrative Framework” to reconcile these heterogeneous findings and provide a roadmap for the future exploration of glymphatic biomarkers.

## 2 Method

This work was conducted in accordance with the PRISMA 2020 statement (Page et al., 2021; checklist reported in Supplementary Table 1) and recorded in PROSPERO with registration number CRD420251266067.

### 2.1 Search strategy

The literature search was performed in three academic electronic databases (Web of Science, PubMed, ScienceDirect) from January 1, 2017, to September 18, 2025 (the initial search was conducted on September 18, 2025). This systematic review investigates the associations between the DTI-ALPS index and AD biomarkers, as well as cognitive outcomes. Specifically, our review addresses the following pivotal questions: Is there a significant association between the DTI-ALPS index and core AD protein biomarkers? If such an association exists, at which stage of AD pathology does glymphatic dysfunction, as reflected by the DTI-ALPS index, first become detectable, and how does it evolve along the AD continuum from cognitively normal through subjective cognitive decline and mild cognitive impairment to AD dementia? Furthermore, through which pathways does this dysfunction influence cognitive function? To achieve these objectives, the eligibility criteria were defined based on the PECOS framework: Participants (individuals across the AD continuum, including preclinical AD, mild cognitive impairment (MCI), and AD dementia, as well as healthy controls); Exposure (assessment of the DTI-ALPS index); Comparator (comparisons between diagnostic groups or continuous variable associations); Outcomes (levels of core AD biomarkers and measures of cognitive performance); and Study Design (cross-sectional or longitudinal studies). According to PECOS criteria, the search strategy was “(‘DTI-ALPS' OR ‘diffusion tensor imaging along the perivascular space' OR ‘ALPS') AND (‘Alzheimer's disease' OR ‘AD' OR ‘Alzheimer').” The search was performed independently by two researchers (Yutong Wu and Chen Zhang), with results cross-verified to ensure comprehensiveness.

### 2.2 Inclusion and exclusion criteria

All original peer-reviewed studies were screened. Study protocols, conference abstracts, editorials, letters to the editor, and dissertations were excluded. The selection was restricted to articles published in English.

The inclusion criteria were the following: (1) original observational studies with cross-sectional or longitudinal designs; (2) included neurobiological assessments (via neuroimaging, CSF, or blood biomarkers) or cognitive evaluations; (3) utilized the DTI-ALPS index to assess glymphatic function; and (4) investigated associations between DTI-ALPS and AD-related biomarkers and/or cognitive outcomes.

### 2.3 Selection and data extraction

Following the removal of duplicates, all articles were screened by two independent reviewers (Y.W. and C.Z.) based on titles and abstracts. To assess eligibility, the full texts of records meeting the inclusion criteria were further evaluated by the same two independent reviewers. Any discrepancies were resolved through consultation with a third reviewer (Z.F.).

Data were extracted by three investigators (Y.W., J.Y., and C.Z.) using a standardized form. The extracted information included the first author, data source, sample size, MRI acquisition details, neuropsychological assessment tools, mean and standard deviation of the DTI-ALPS index across different subgroups, correlation or regression coefficients between the DTI-ALPS and AD-related biomarkers, main findings, and study novelty.

### 2.4 Quality assessment

The methodological quality and risk of bias of the included studies were independently assessed by two researchers (Y.W. and J.Y.) using the Newcastle-Ottawa Scale (NOS; reported in Supplementary Table 2). The scale evaluates studies on three domains: selection, comparability, and outcome. Discrepancies between reviewers were resolved through discussion and consensus. If a disagreement persisted, a third researcher (Z.F.) was engaged to provide a final decision.

To maintain the transparency and minimize potential bias in our own review process, we also evaluated the risk of bias of the systematic review itself, following its completion. This was achieved using the Risk of Bias in Systematic Reviews (ROBIS) tool (Whiting et al., 2016; reported in Supplementary Tables 3–7). Two authors (Y.W. and J.Y.) independently scored the four ROBIS domains (eligibility, identification, data collection/appraisal, and synthesis) to minimize potential bias.

### 2.5 Data synthesis

A meta-analysis was conducted for data demonstrating sufficient homogeneity in methodology and outcome assessment types, while a narrative synthesis was performed for studies not suitable for quantitative synthesis.

Random-effects meta-analyses were employed to estimate the effect sizes of differences in the DTI-ALPS index between subgroups. Additionally, we estimated the pooled correlation coefficients between the DTI-ALPS index and specific neurobiological biomarkers (e.g., Aβ PET deposition, tau PET deposition) as well as cognitive measures (e.g., MMSE, MoCA). Inter-study heterogeneity was assessed using Cochran's Q test and quantified via the *I*^2^ statistic (Higgins and Thompson, 2002). Publication bias was assessed using funnel plots, Egger's test when *k* ≥ 10, and trim-and-fill sensitivity analyses. Furthermore, to explore sources of heterogeneity, the following moderators were evaluated: study cohort source (public datasets vs. clinical datasets), PET tracers, number of diffusion gradient directions, b-values, mean age difference between subgroups, difference in age standard deviation, regions of interest (ROIs) placement method (automated script vs. manual), ROI size, DTI fitting software, female ratio and number of corrected covariates. All meta-analyses were performed using the meta package in R statistical software (Schwarzer et al., 2015).

## 3 Results

### 3.1 Study selection

**Figure 1:**
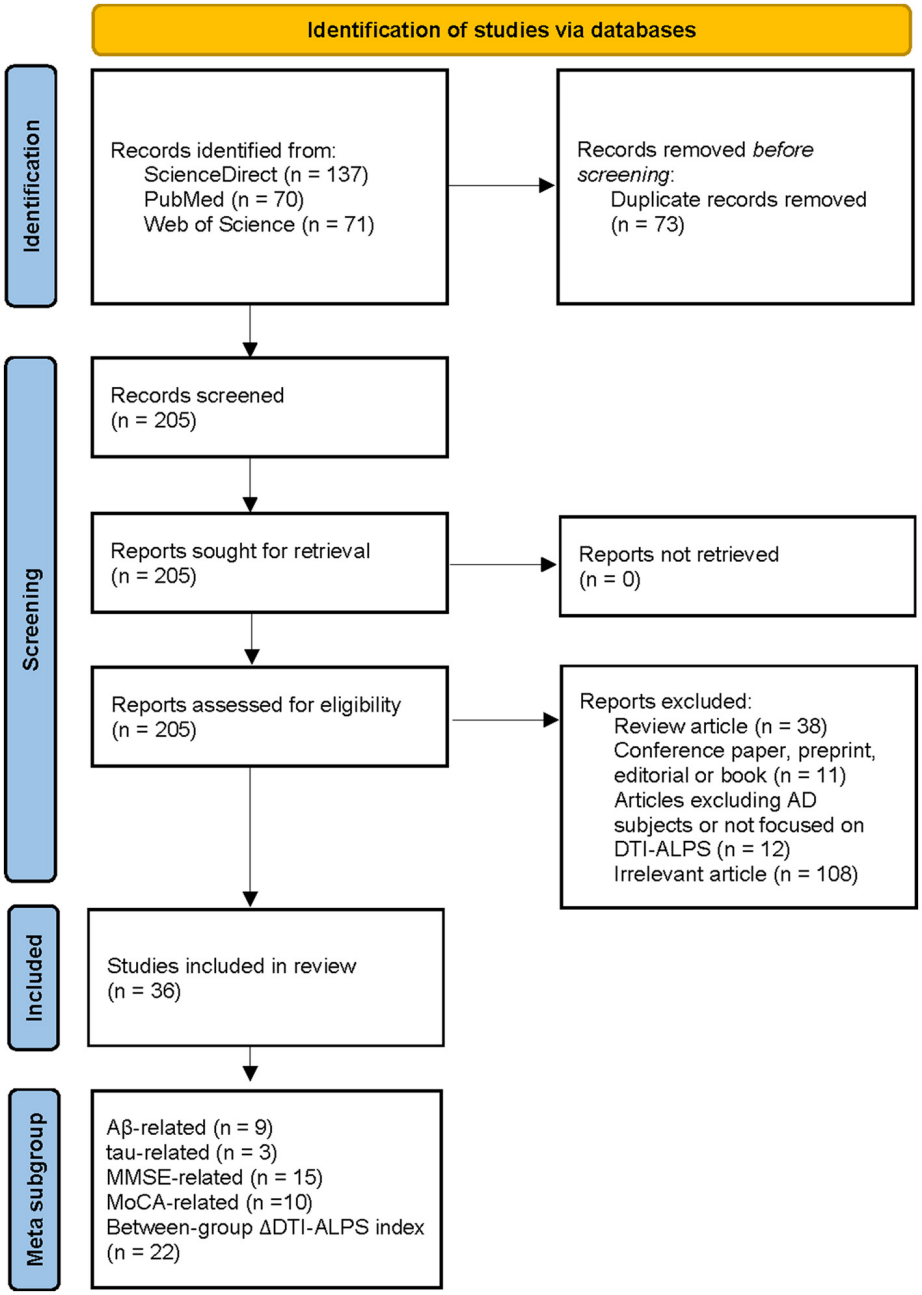
The flow of literature screening for inclusion and exclusion. Adapted from the PRISMA 2020 flow diagram template (Page et al., 2021), licensed under CC BY 4.0.

A total of 278 records were initially identified through the search strategy. Following the removal of duplicates, articles were screened by title and abstract, resulting in the exclusion of review articles (*n* = 38), conference papers, preprints, editorials, or books (*n* = 11), and studies on irrelevant topics (*n* = 108). The remaining 48 studies underwent full-text assessment. Twelve articles were subsequently excluded because they did not include participants with AD or lacked data on the DTI-ALPS index. Ultimately, 36 studies met the eligibility criteria and were included in this systematic review. The study selection process is illustrated in Figure 1.

### 3.2 Characteristics of the selected studies

Key characteristics of the 36 included studies are summarized in Table 1. The majority employed a cross-sectional design (*n* = 27), while nine utilized a longitudinal approach. Based on the NOS assessment, the overall methodological quality of the studies was high. Thirteen studies utilized public datasets (e.g., ADNI, UKB, and AIBL), and 27 involved clinical cohorts, some studies included more than one data source. Sample sizes ranged from 16 to 36,469, with 13 studies including fewer than 100 participants. Most studies involved patients with MCI (*n* = 18). Additionally, five studies included participants with Subjective cognitive decline (SCD) or subjective memory concern. Meta-analyses (Table 2) revealed significant correlations of the DTI-ALPS index (*N* = 22) with amyloid-beta PET deposition (*N* = 9), MMSE scores (*N* = 15), and MoCA scores (*N* = 10). However, the association with tau PET deposition (*N* = 3) was non-significant after adjusting for publication bias.

**Table 1:** Key information included in the literature.

References	Data source	MRI modalities	Neuropsychological assessments	Number of subjects	Important results	Novelty/contribution
Taoka et al. (2017)	Department of Radiology, Nagoya University	DTI	MMSE	AD (16), MCI (9), SCD (6)	Demonstrated a significant positive correlation between the DTI-ALPS index and MMSE scores.	Introduced the DTI-ALPS method to non-invasively assess GS function.
Steward et al. (2021)	AIBL; VEL015 trial	DTI, SWI, T1, T2	MMSE, ADAS-Cog11, MAC-Q, CERAD	AIBL: CN (10), MCI (10)	Revealed a lower right-hemisphere DTI-ALPS index in AD and MCI groups compared to controls, which correlated with worse cognitive performance (MMSE and ADAS-Cog11).	First to validate the DTI-ALPS index in a dementia-risk cohort and utilized multimodal MRI to refine ROI placement.
				VEL015: AD (16)		
Ota et al. (2022)	National Center of Neurology and Psychiatry	T1, DTI, PET	MMSE, CDR, MoCA	AD (21), CN (36)	Identified significant negative correlations between the DTI-ALPS index and brain Aβ (^11^C-PiB) and tau/neuroinflammation (^18^8F-THK5351) PET SUVRs.	First to investigate the direct association between the DTI-ALPS index and *in vivo* Aβ and tau pathologies using PET imaging.
Hsu et al. (2023)	Linkou Chang Gung Memorial Hospital	PET, DTI	MMSE, CDR, CERAD	AD (37), CN (13)	Established the DTI-ALPS index as a significant mediator of the relationship between Aβ/tau pathology and cognitive dysfunction.	Provided the first evidence using mediation analysis that glymphatic dysfunction acts as a link between Aβ/tau pathology and cognitive impairment.
Liang et al. (2023)	Department of Neurology, China-Japan Friendship Hospital	DTI	MMSE, MoCA	AD (38), MCI (18), VCI (21), CN (28)	Found a significantly lower DTI-ALPS index in AD, MCI, and VCI groups compared to controls, which positively correlated with cognitive scores (MMSE and MoCA).	Conducted the first comparative study of the DTI-ALPS index across dementia etiologies, including AD, MCI, and vascular cognitive impairment (VCI).
Chang et al. (2023)	Kaohsiung Chang Gung Memorial Hospital	T1, DTI	MMSE, CASI	EOAD (130), CLT (137)	Showed that in young-onset AD, a lower DTI-ALPS index was associated with cognitive deficits, a relationship fully mediated by gray matter volume in DTI-ALPS-coherent regions.	First investigation of the DTI-ALPS index in EOAD, revealing that gray matter integrity mediates its link to cognition.
Saito et al. 2023a)	ADNI	DTI, PET	MMSE, MoCA, CDR, ADAS, RAVLT, TMT-B, Ecog, LDELTOTAL, TRABSCOR, FAQ	CN (82), AD (45)	Demonstrated that COMBAT harmonization effectively reduced interscanner variability, strengthening the observed group differences and biomarker correlations of the DTI-ALPS index.	First to validate the use of COMBAT harmonization for reducing interscanner variability in multisite DTI-ALPS studies.
Huang et al. (2024)	ADNI; UKB	DTI, PET	MMSE, CDR, PACC, ADNI-Mem, ADNI-EF	ADNI: AD (47), MCI (137), CN (235; According to the A/T/N status classification)	Reported that a lower baseline DTI-ALPS index predicted accelerated Aβ accumulation and clinical progression, with its effect on cognitive decline being fully mediated by Aβ and brain atrophy. Furthermore, Glymphatic failure may precede amyloid pathology.	Provided the first longitudinal evidence from large-scale cohorts (ADNI and UKB) that glymphatic dysfunction predicts future AD pathology and clinical progression.
				UKB: CN (36050)		
Zhang et al. (2024)	China National Clinical Research Center Alzheimer's Disease and Neurodegenerative Disorder Research	T1, T2, DTI	CDR, MMSE, MoCA, AQ, RAVLT, TMT-A, TMT-B, CDT, BNT, DST, SDMT, NPI	AD (15), MCI (15), CN (26)	Revealed a negative correlation between the DTI-ALPS index and PVS volume fraction, and linked a lower index to deficits in specific cognitive domains.	First to link the DTI-ALPS index with a quantitative PVS volume fraction and detailed, domain-specific cognitive functions.
Li et al. (2024)	SILCODE, ADNI	DTI, PET	MoCA, MMSE, SCD-9, FAQ	SILCODE: CN (215), SCD (194), MCI (153), AD (92)	Indicated that glymphatic dysfunction, measured by the DTI-ALPS index, is present at the subjective cognitive decline (SCD) stage and predicts cognitive progression.	First to demonstrate glymphatic impairment at the SCD stage and validate the DTI-ALPS index's performance across both Asian and Western cohorts.
				ADNI: CN (197), SCD (191), MCI (158), AD (104)		
Hong et al. (2024)	ADNI	PET, DTI, T1, T2	MMSE, ADNI-Mem, ADNI-EF, ADNI-Lan, ADNI-VS	CN- (Aβ-, 40), CN+ (Aβ+, 48), MCI+ (Aβ+, 26), AD+ (Aβ-, 19)	Showed that the DTI-ALPS index mediated the effects of both Aβ accumulation and white matter hyperintensity (WMH) burden on memory and language performance.	Simultaneously, evaluated the contributions of Aβ pathology and cerebral small vessel disease (WMH) to glymphatic dysfunction.
Sacchi et al. (2024)	Fondazione IRCCS Ca' Granda Ospedale Maggiore Policlinico in Milan	T1, FLAIR, multishell DW-MRI	MMSE	AD (47), MCI (17), CN (23)	Observed a lower DTI-ALPS index in more atrophic AD patients, alongside increased PVS, suggesting its utility in reflecting advanced neurodegenerative stages.	Introduced a comprehensive, multicompartment assessment of the GS using a combination of neuroimaging and CSF biomarkers.
Shang et al. (2024)	The First Affiliated Hospital of Soochow University	DTI, T1, T2	MMSE, MoCA, PSQI	ADSD (40), ADNSD (39), CN (25)	Found that the DTI-ALPS index partially mediated the association between sleep disturbances and cognitive impairment in AD patients.	First to use the DTI-ALPS index to provide direct evidence linking sleep disturbances to impaired glymphatic function in AD.
Kim et al. (2024)	Seoul National University Bundang Hospital	DTI, T1, PET	CERAD	AD (65), CN (15)	Confirmed a lower DTI-ALPS index in AD, which was associated with regional brain atrophy and cognitive decline, independent of Aβ PET SUVRs.	Demonstrated that the DTI-ALPS index's association with neurodegeneration is independent of cerebral Aβ burden.
Okazawa et al. (2024)	Biomedical Imaging Research Center, University of Fukui	PET, T1, T2, FLAIR, DTI	MMSE	MCI & DAT (56), CN (27)	Reported a robust linear correlation between the DTI-ALPS index and global Aβ burden quantified using the centiloid scale.	First to apply the centiloid scale for a standardized, quantitative assessment of the DTI-ALPS index's relationship with global Aβ deposition.
Xia et al. (2025)	ADNI	T1, DTI	MMSE	SCD (344), MCI (761), AD (246)	Found a lower DTI-ALPS index in AD, which correlated with choroid plexus enlargement and markers of white matter microstructural damage (PSMD).	Investigated the combined roles of choroid plexus volume, white matter damage, and systemic inflammation in relation to glymphatic function in AD.
Zhang Y. et al. (2025)	Ren Ji Hospital	PET, T1, DTI	MMSE, MoCA	EOAD (37), LOAD (62), MCI (35), SCD (6)	Showed that Aβ pathology, but not tau, fully mediated the effects of the DTI-ALPS index on brain metabolism (FDG-PET) and cognition.	First to use causal mediation analysis with trimodal PET to specify Aβ, not tau, as the key mediator between glymphatic function and cognitive decline.
Schirge et al. (2025)	ActiGliA, DELCODE, ADNI	DTI, FLAIR	MMSE, CDR	ActiGliA: AD (16), CN (18)	Demonstrated in three independent cohorts that a lower DTI-ALPS index was associated with lower CSF Aβ concentration, higher WMH burden, and worse cognitive scores.	Validated the associations of the DTI-ALPS index with CSF Aβ, WMH burden, and cognition across three large, independent cohorts.
				DELCODE: AD (54), CN (67)		
				ADNI: AD (43), CN (49)		
Li et al. (2025)	National Human Brain Bank for Health and Disease; ADNI	DTI, PET	PACC	National Human Brain Bank for Health and Disease (postmortem samples):Aβ- (9), Aβ+ (9)	Revealed that the association between the DTI-ALPS index and Aβ pathology was significantly attenuated after correcting for white matter microstructural bias using postmortem data.	Developed and validated a corrected DTI-ALPS index (cALPS) using postmortem brain DTI to minimize bias from white matter microstructure.
				ADNI: Aβ- (49), Aβ+ (61)		
Chen Z. et al. (2025)	Xuanwu Hospital, Capital Medical University	T1, DTI	MMSE, MoCA	AD (31), CN (24)	Identified hippocampal PVS as a mediator between retinal vascular parameters and cognitive impairment, and linked these changes to a reduced DTI-ALPS index.	First to integrate retinal vascular imaging with brain glymphatic markers, proposing an “eye-brain axis” in AD pathophysiology.
You et al. 2025a)	Korea University Anam Hospital	DTI, 2D phase-contrast MRI	MMSE, SNSB-II	CN (50), CN aging (46), AD (64)	Established a negative correlation between the DTI-ALPS index and cerebral arterial pulsatility, suggesting vascular stiffness impairs glymphatic function.	Introduced a novel, accessible 2D phase-contrast MRI method to measure cerebral arterial pulsatility as a driver of glymphatic function.
Luo et al. (2025)	ADNI	T1, DTI, T2, PET	MMSE, CDR, Neuropsychological Summary Scores	EOAD (38), LOAD (60), CN (Age < 65, 40), CN (Age>=65, 69)	Uncovered that a lower DTI-ALPS index was primarily associated with Aβ in early-onset AD, but with age and WMH in late-onset AD.	First to use the DTI-ALPS index to compare glymphatic dysfunction between early- and late-onset AD, revealing distinct underlying pathological associations.
Zhang Q. et al. (2025)	The Second Affiliated Hospital of Soochow University	T1, DTI	MoCA, MMSE	CN (28), MCI (19), Mild AD (17), Moderate AD (25)	Found that a lower DTI-ALPS index was associated with widespread cortical thinning and atrophy, particularly in moderate AD stages.	Systematically correlated the DTI-ALPS index with a comprehensive set of cortical metrics across different stages of AD severity.
Wang et al. (2025)	ADNI	T1, DTI, PET	MMSE, ADNI-Mem, ADNI-EF	IHD (159), non-IHD (1226)	Linked ischemic heart disease (IHD) to impaired glymphatic function and showed that free water (FW), not DTI-ALPS, mediated the effect of IHD on cognitive decline.	First to investigate the link between ischemic heart disease (IHD) and brain glymphatic MRI indices, proposing a new mechanism for IHD-associated AD risk.
Firbank et al. (2025)	Translational and Clinical Research Institute, Newcastle University	DTI, T1, FLAIR, PET	ACE-R, MMSE	DLB (32), AD (14), MCI-LB (31), MCI-AD (31), CN (48)	Showed that a lower DTI-ALPS index in Lewy body diseases was associated with cognitive decline and elevated plasma markers of neuronal injury (NfL) and astrocytosis (GFAP).	First systematic evaluation of the DTI-ALPS index in Lewy body diseases and its association with plasma biomarkers of neurodegeneration.
Jiao et al. (2025)	The First Affiliated Hospital of Harbin Medical University	PET, DTI, T1, T2	MMSE, MoCA, CDR, HAMD, ADL, CDT	CN (19), PAD (16), AD (18)	Reported that the DTI-ALPS index mediated the relationship between cerebral perfusion deficits (early-phase Aβ PET) and cognitive impairment.	Utilized simultaneous PET/MR to provide an integrated, single-session analysis of perfusion, glymphatic function, and vascular burden in AD.
Guo et al. (2025)	ADNI, CTPCS-HEAD	DTI, T1	MMSE, CDR, FAQ	ADNI: CN (35), SMC (28), MCI (82), AD (35)	Validated in two independent cohorts that a lower DTI-ALPS index was associated with AD diagnosis, longitudinal cognitive decline, and increased risk of clinical conversion.	Validated the diagnostic and prognostic value of the DTI-ALPS index for AD dementia and cognitive decline across two independent clinical cohorts.
				CTPCS-HEAD: CN (25), SMC (51), MCI (32), AD (19)		
Chang et al. (2025)	Kaohsiung Chang Gung Memorial Hospital	T1, DTI, PET	MMSE, CASI	AD (157), CN (117)	Found that tau burden was a stronger predictor of cognitive decline than DTI-ALPS, with astrocytic activation (GFAP) mediating the tau-atrophy link.	Disentangled the distinct roles of tau pathology, astrocytic activation (GFAP), and glymphatic function in predicting neurodegeneration and cognitive decline.
Agah et al. (2025)	University of California, Davis Alzheimer's Disease Research Center	T1, FLAIR, DTI	SENAS, EF, EM	CN (359), MCI (156), AD (59)	Revealed through Bayesian modeling that DTI-ALPS had limited unique predictive power for cognitive change when considered alongside other markers like FW and WMH.	First to use Bayesian model averaging to quantify the unique vs. redundant contributions of DTI-ALPS among multiple white matter integrity markers.
Jungwon et al. (2025)	Chungbuk National University Hospital	DTI	CDR, MMSE	AD (16)	Demonstrated that a higher baseline DTI-ALPS index predicted less cognitive decline over a 1-year follow-up in AD patients.	Provided longitudinal evidence for the predictive value of the baseline DTI-ALPS index on 1-year cognitive decline in an AD-specific cohort.
Wu et al. (in press)	UKB	DTI	NM, RT, TMT, SDST	CN (31,579)	Provided genetic evidence through GWAS and Mendelian randomization that genetically predicted lower DTI-ALPS index causally increases the risk for AD.	Conducted the first GWAS of the DTI-ALPS index and provided the first genetic evidence for a causal link between glymphatic dysfunction and AD risk.
You et al. 2025b)	Korea University Anam Hospital	FLAIR, T2, T1, SWI, DTI	SNSB-II, CDR, MMSE	SCI (106), early MCI (66), late MCI (87), AD (100)	Developed a diagnostic tree analysis model showing that the DTI-ALPS index, combined with other MRI markers, significantly improved prediction of late-stage cognitive impairment.	Developed an intuitive Decision Tree Analysis (DTA) model integrating DTI-ALPS and other MRI markers for improved diagnostic accuracy.
Yu et al. (2025)	The First Affiliated Hospital of Nanjing Medical University	DTI	MMSE, MoCA	CN (18), MCI (20), AD (22)	Proposed that hippocampal microstructural damage precedes significant DTI-ALPS decline, suggesting distinct temporal trajectories for these biomarkers in AD progression.	Proposed a novel multimodal biomarker framework revealing distinct temporal progression patterns for DTI-ALPS vs. hippocampal diffusivity in AD.
Lin et al. (2025)	The First Affiliated Hospital of Nanjing Medical University	T1, DTI	MMSE, MoCA, CDR, STT-A, STT-B, PSQI, VFT, BNT, MES, HAMD	AD (28), MCI (40), SCD (66), CN (54)	Showed that cerebrospinal fluid (CSF) volume mediated the association between choroid plexus enlargement and glymphatic dysfunction (lower DTI-ALPS index).	First to provide evidence that CSF volume mediates the relationship between choroid plexus enlargement and glymphatic dysfunction.
Bao et al. (2025)	ADNI	DTI	ADNI_Mem, ADNI_EF, ADNI_Lan, ADNI_VS	CN (253, 30 to MCI), MCI (266, 73 to AD)	Found a higher DTI-ALPS index was associated with delayed MCI/AD progression, an effect partially mediated by Aβ/tau pathology and executive function.	First to quantify the potential delay (approx. 3.5 years) in MCI/AD progression associated with a higher DTI-ALPS index.
Chen F. et al. (2025)	Zhejiang Provincial People's Hospital	T1, T2, FLAIR, DTI	MMSE, MoCA	AD (89), aMCI (24), CN (32)	Associated a lower DTI-ALPS index with a greater volume and number of enlarged PVS (EPVS) quantified via a deep learning model.	Applied a deep learning model (VB-Net) for automated segmentation of EPVS to correlate with the DTI-ALPS index.

The abbreviations appearing in the table correspond to: Data source (AIBL, Australian Imaging Biomarkers and Lifestyle Study of Aging; ADNI, Alzheimer's disease neuroimaging initiative; UKB, UK biobank; SILCODE, Sino longitudinal study on cognitive decline; ActiGliA, Activity of cerebral networks, amyloid and microglia in aging; DELCODE, German center for neurodegenerative diseases longitudinal cognitive impairment and dementia; CTPCS-HEAD, Chinese tropical population cohort study on healthy aging and dementia), Neuropsychological assessments (MMSE, Mini mental state examinations; ADAS-Cog11, Alzheimer's disease assessment scale–cognitive subscale; MAC-Q, Memory complaint questionnaire; CERAD, Consortium to establish a registry for Alzheimer's disease; CDR, Clinical dementia rating; MoCA, Montreal cognitive assessment; CASI, Cognitive abilities screening instrument; ADAS, Alzheimer's disease assessment scale; RAVLT, Rey auditory verbal learning test; TMT, Trail making test; ECog, Everyday cognition; LDELTOTAL, Logical memory delayed recall total; TRABSCOR, Time to complete part B of the Trail Making Test score; FAQ, Functional activities questionnaire; PACC, Preclinical Alzheimer's cognitive composite; ADNI-Mem, Memory function; ADNI-EF, Executive function; ADNI-Lan, Language; ADNI-VS, Visuospatial function; AQ, Alzheimer's questionnaire; CDT, Clock drawing test; BNT, Boston naming test; DST, Digit span test; SDMT, Symbol digit modalities test; NPI, Neuropsychiatry inventory; SCD-9, 9-item version of the subjective cognitive decline questionnaire; PSQI, Pittsburgh sleep quality index; SNSB-II, Seoul neuropsychological screening battery-II; ACE-R, Addenbrookes Cognitive Examination-Revised; HAMD, Hamilton depression scale; ADL, Activity of daily living; SENAS, Spanish and English neuropsychological assessment scales; EF, Executive function; EM, Episodic memory; NM, Numeric memory test; RT, Reaction time; SDST, Symbol digit substitution test; STT, Shape trail test; VFT, Verbal fluency test; MES, Memory and executive screening), and Number of subjects (AD, Alzheimer's disease; MCI, Mild cognitive impairment; SCI, Subjective cognitive impairment; CN, Cognitively normal; VCI, Vascular cognitive impairment; EOAD, Early-onset AD; LOAD, Late-onset AD; CLT, Age-matched control; SCD, Subjective cognitive decline; ADSD, AD patients with sleep disorders; ADNSD, AD patients without sleep disorders; DAT, Early Alzheimer's type dementia; IHD, Ischemic heart disease; DLB, Dementia with Lewy bodies; MCI-LB, MCI with Lewy bodies; PAD, Prodromal Alzheimer's disease; SMC, Subjective memory concern).

**Table 2:** Comprehensive meta-analytic results of the DTI-ALPS index: group differences across the Alzheimer's disease continuum and correlations with biomarkers.

Comparison	*k*	N		ES_type	ES	95% CI	*I* ^2^	tau^2^	Egger_p	TF_k	TF_ES	95% TF_CI
		N_case	N_control									
AD vs. CN M	18	1,031	1,359	SMD	**−0.862** ^***^	[−1.054, −0.67]	0.722	0.152	< 0.001	10	**−0.555** ^***^	[−0.794, −0.316]
AD vs. CN L	9	498	497	SMD	**−0.718** ^***^	[−0.999, −0.44]	0.745	0.222	0.090	5	**−0.402** ^*^	[−0.753, −0.052]
AD vs. CN R	9	498	497	SMD	**−0.797** ^***^	[−1.044, −0.55]	0.661	0.152	0.271	0		
AD vs. MCI M	10	662	1,232	SMD	**−0.618** ^**^	[−0.991, −0.24]	0.789	0.298	0.367	1	−0.46	[−0.978, 0.059]
AD vs. MCI L	4	142	69	SMD	−0.765	[−1.61, 0.081]	0.811	0.621	—	0		
AD vs. MCI R	4	142	69	SMD	−0.981	[−2.11, 0.149]	0.875	1.194	—	0		
MCI vs. CN M	9	467	820	SMD	**−0.284** ^***^	[−0.4, −0.17]	0.117	0	—	4	**−0.212** ^***^	[−0.319, −0.105]
MCI vs. CN L	4	69	86	SMD	**−0.546** ^**^	[−0.872, −0.22]	0.000	0	—	1	**−0.588** ^***^	[−0.894, −0.283]
MCI vs. CN R	4	69	86	SMD	**−0.385** ^*^	[−0.708, −0.06]	0.000	0	—	0		
DTI-ALPS-Aβ PET (partial)	6	1,045		r	**−0.343** ^***^	[−0.448, −0.23]	0.647	0.014	—	2	**−0.284** ^***^	[−0.409, −0.148]
DTI-ALPS-Aβ PET (corr)	3	526		r	**−0.386** ^***^	[−0.543, −0.2]	0.797	0.043	—	0		
DTI-ALPS-tau PET (partial)	3	526		r	**−0.311** ^*^	[−0.518, −0.07]	0.778	0.037	—	2	−0.14	[−0.399, 0.139]
DTI-ALPS-MMSE (partial)	8	872		r	**0.379** ^***^	[0.193, 0.538]	0.883	0.083	—	2	**0.307** ^**^	[0.121, 0.473]
DTI-ALPS-MMSE (corr)	7	702		r	**0.429** ^***^	[0.304, 0.539]	0.697	0.029	—	3	**0.321** ^***^	[0.154, 0.470]
DTI-ALPS-MoCA (partial)	4	1,104		r	**0.409** ^***^	[0.192, 0.588]	0.926	0.067	—	2	0.224	[−0.087, 0.495]
DTI-ALPS-MoCA (corr)	6	536		r	**0.407** ^***^	[0.265, 0.532]	0.664	0.026	—	3	**0.283** ^**^	[0.102, 0.447]

AD, Alzheimer's disease; CN, Cognitively Normal; MCI, mild cognitive impairment; DTI-ALPS, diffusion tensor image analysis along the perivascular space; Aβ, β-amyloid; k, number of independent studies; N_case/N_control, sample size of case/control group; ES_type, type of effect size; SMD, standardized mean difference; r, correlation coefficient; 95% CI, 95% confidence interval of the effect size; I^2^, proportion of heterogeneity; tau^2^, between-study variance; Egger_p, p-value from Egger's linear regression test for publication bias (calculated only when k ≥ 10); TF_k, number of missing studies estimated by the Trim and Fill method; TF_ES, Trim-and-Fill-adjusted pooled effect size; 95% TF_CI, 95% confidence interval of the adjusted effect size. ^*^p < 0.05; ^**^p < 0.01; ^***^p < 0.001. Bold values indicate statistically significant pooled effect estimates, defined as estimates with 95% confidence intervals that do not include the null value of zero.

### 3.3 Multimodal associations between the DTI-ALPS index and core biomarkers of AD

#### 3.3.1 Associations with core pathological proteins (A/T)

**Figure 2:**
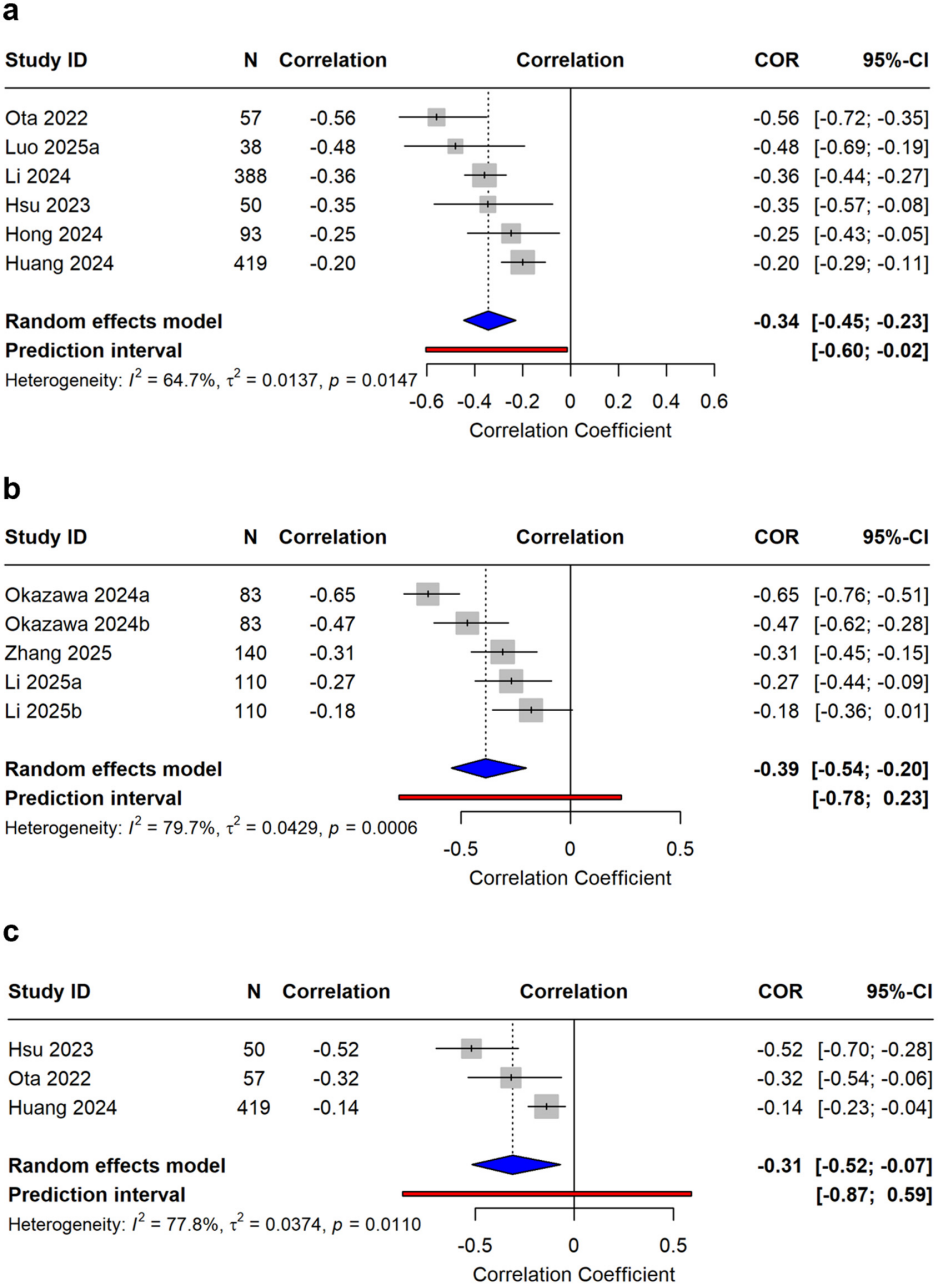
Forest plots illustrating the associations between the DTI-ALPS index and core AD protein biomarkers. **(a)** Studies using partial correlation analysis to assess the association between the DTI-ALPS index and Aβ PET deposition. **(b)** Studies using correlation analysis to assess the association between the DTI-ALPS index and Aβ PET deposition. **(c)** Studies using partial correlation analysis to assess the association between the DTI-ALPS index and tau PET deposition.

Twenty studies investigated the association between the DTI-ALPS index and Aβ biomarkers, including Aβ PET deposition (Ota et al., 2022; Hsu et al., 2023; Saito et al., 2023a; Hong et al., 2024; Kim et al., 2024; Li et al., 2024, 2025; Okazawa et al., 2024; Bao et al., 2025; Chang et al., 2025; Firbank et al., 2025; Jiao et al., 2025; Luo et al., 2025; Zhang Y. et al., 2025), CSF Aβ (Huang et al., 2024; Sacchi et al., 2024; Bao et al., 2025; Guo et al., 2025; Schirge et al., 2025; Yu et al., 2025), and plasma markers (Firbank et al., 2025; Lin et al., 2025). Of these, 15 were cross-sectional and five were longitudinal studies. A substantial body of evidence supports a link between glymphatic dysfunction and amyloid pathology. Cross-sectional studies have consistently demonstrated that a lower DTI-ALPS index correlates with higher cerebral Aβ PET burden (Ota et al., 2022; Hsu et al., 2023; Okazawa et al., 2024; Zhang Y. et al., 2025), a relationship that appears particularly pronounced in early-onset AD (Luo et al., 2025). These neuroimaging findings are corroborated by fluid biomarker studies, which report significant associations between reduced DTI-ALPS index and pathological levels of CSF Aβ42 (Huang et al., 2024; Sacchi et al., 2024; Guo et al., 2025; Schirge et al., 2025; Yu et al., 2025). Longitudinal investigations further suggest that baseline DTI-ALPS abnormalities may precede overt amyloid accumulation (Huang et al., 2024). Our meta-analysis reinforces these qualitative findings. Across six studies utilizing partial correlation (Figure 2a), we observed a significant negative association between the DTI-ALPS index and Aβ PET deposition (pooled correlation = −0.34, 95% CI: −0.45 to −0.23, *I*^2^ = 64.7%). Notably, this result remained robust even after adjusting for potential publication bias (adjusted pooled correlation = −0.28, 95% CI: −0.41 to −0.15, *p* < 0.0001; Supplementary Figures 3a, b). A similar negative association was also observed in studies using correlation analysis (Figure 2b; pooled correlation = −0.39, 95% CI: −0.54 to −0.20, *I*^2^ = 79.7%).

Despite the robust statistical correlation, the biological specificity of this link remains debated. Some studies failed to replicate the association after strictly controlling for covariates (Kim et al., 2024; Firbank et al., 2025), and recent postmortem validation suggests that the DTI-ALPS-Aβ correlation may be confounded by white matter microstructural degeneration rather than reflecting pure fluid dynamics (Li et al., 2025). Consistent with this, exploratory meta-regression suggested that heterogeneity was associated with the number of adjusted covariates (*R*^2^ = 99.97%, *p* = 0.0014), female ratio (*R*^2^ = 85.74%, *p* = 0.0005) , DTI fitting software (*R*^2^ = 84.99%, *p* = 0.0062) and PET tracer type (18F-AV45 vs. 11C-PiB; *R*^2^ = 58.64%, *p* = 0.0377).

Only ten studies investigated the association between the DTI-ALPS index and tau biomarkers, including tau PET deposition (Ota et al., 2022; Hsu et al., 2023; Huang et al., 2024; Bao et al., 2025; Chang et al., 2025; Zhang Y. et al., 2025), CSF tau (Huang et al., 2024; Bao et al., 2025; Guo et al., 2025; Yu et al., 2025), and plasma markers (Firbank et al., 2025; Lin et al., 2025). Of these, six were cross-sectional and four were longitudinal studies. Distinct from Aβ, the relationship between the DTI-ALPS index and tau pathology appears notably more robust within neuroimaging contexts. Six studies investigating the association with tau PET deposition consistently reported negative correlations (Ota et al., 2022; Hsu et al., 2023; Huang et al., 2024; Bao et al., 2025; Chang et al., 2025; Zhang Y. et al., 2025). However, this consistency does not extend to fluid biomarkers, where results remain equivocal. For instance, Huang et al. (2024) reported no significant correlations between the DTI-ALPS index and CSF p-tau181 or t-tau levels, a lack of association that was subsequently corroborated by Guo et al. (2025). Our quantitative synthesis mirrors this ambiguity (Figure 2c). Although the initial random-effects model suggested a significant negative correlation (pooled correlation = −0.31, 95% CI: −0.52 to −0.07, *I*^2^ = 77.8%), the association was rendered non-significant after adjusting for publication bias (adjusted pooled correlation = −0.14, 95% CI: −0.40 to 0.14, *p* = 0.3256; Supplementary Figure 3c). Collectively, current evidence is insufficient to establish DTI-ALPS as a direct correlate of tau pathology.

#### 3.3.2 Associations with neurodegeneration (N)

In contrast to its complex relationship with core pathological proteins, the associations between the DTI-ALPS index and neurodegeneration, as well as brain structural health indicators, demonstrate high consistency and robustness. Numerous studies have confirmed that a decreased DTI-ALPS index is significantly correlated with widespread brain atrophy, including reduced total gray matter volume (Hsu et al., 2023); atrophy in AD-typical regions such as the hippocampus and entorhinal cortex (Kim et al., 2024; Okazawa et al., 2024); and cortical thinning in multiple brain regions (Zhang Q. et al., 2025).

Moreover, the DTI-ALPS index is closely associated with other indicators reflecting brain parenchymal health. For example, it is positively correlated with cerebral perfusion levels (Jiao et al., 2025) and is linked to markers of cerebral small vessel disease (CSVD) and barrier dysfunction, such as white matter hyperintensity (WMH) burden, enlarged PVS, increased choroid plexus volume, and white matter microstructural damage (Hong et al., 2024; Sacchi et al., 2024; Zhang et al., 2024; Xia et al., 2025).

#### 3.3.3 Associations with cognitive impairment (C)

Thirty-three studies investigated the association between the DTI-ALPS index and cognitive function. Of these, 26 were cross-sectional and seven were longitudinal studies. Pioneering validation studies by Taoka et al. (2017) and Liang et al. (2023) first established the clinical relevance of this metric, demonstrating robust positive correlations between a higher DTI-ALPS index and better scores on global cognitive assessments, such as the MMSE and MoCA. This relationship extends to domain-specific cognitive functions, with a lower index linked to deficits in memory, executive function, and processing speed, which are core cognitive domains affected in AD (Hong et al., 2024; Huang et al., 2024; Zhang et al., 2024).

**Figure 3:**
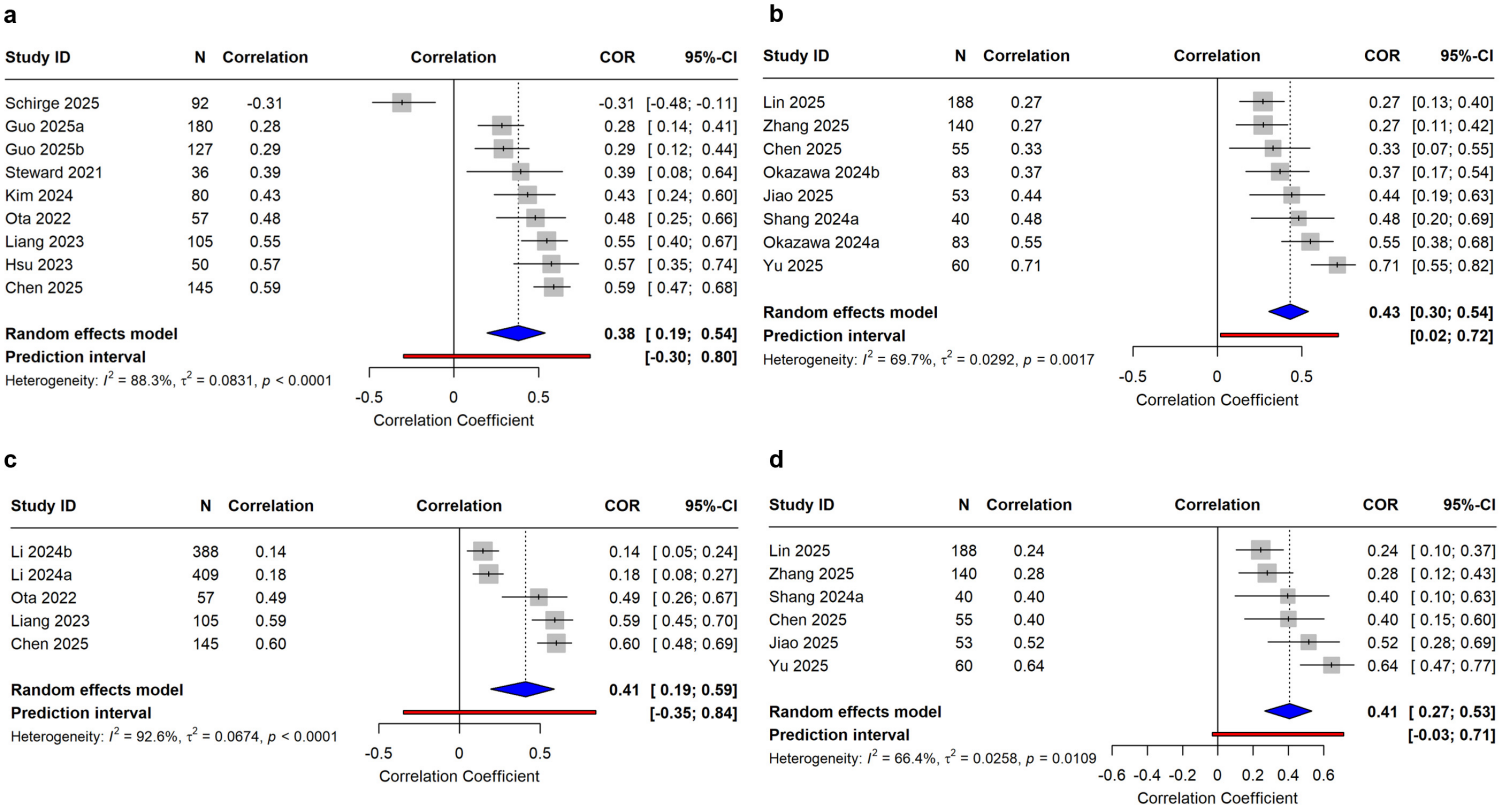
Forest plots illustrating the associations between the DTI-ALPS index and cognitive function. **(a)** Studies using partial correlation analysis to assess the association between the DTI-ALPS index and MMSE scores. **(b)** Studies using correlation analysis to assess the association between the DTI-ALPS index and MMSE scores. **(c)** Studies using partial correlation analysis to assess the association between the DTI-ALPS index and MoCA scores. **(d)** Studies using correlation analysis to assess the association between the DTI-ALPS index and MoCA scores.

Random-effects models demonstrated significant positive correlations between the DTI-ALPS index and MMSE scores (partial pooled r = 0.38, 95% CI: 0.19 to 0.54, *I*^2^ = 88.3%; correlation pooled *r* = 0.43, 95% CI: 0.30 to 0.54, *I*^2^ = 69.7%; Figures 3a, b). Similarly, regarding the association with MoCA scores, random-effects models revealed significant positive correlations (partial pooled *r* = 0.41, 95% CI: 0.19 to 0.59, *I*^2^ = 92.6%; correlation pooled *r* = 0.41, 95% CI: 0.27 to 0.53, *I*^2^ = 66.4%; Figures 3c, d). Although tests for publication bias suggested potential asymmetry, the associations remained statistically significant after adjustment, with adjusted correlations of 0.31 (partial, *p* = 0.0015) and 0.32 (correlation, *p* = 0.0002) for MMSE, 0.22 (partial, *p* = 0.1561) and 0.28 (correlation, *p* = 0.0025) for MoCA (Supplementary Figure 4). Notably, our meta-regression identified that inter-study heterogeneity was largely driven by methodological factors. Specifically, the study cohort source (public datasets vs. clinical datasets; MMSE partial *R*^2^ = 53.56%, *p* = 0.0050; MoCA partial *R*^2^ = 100.00%, *p* < 0.0001), ROI size (MoCA partial *R*^2^ = 100.00%, *p* < 0.0001), DTI fitting software (FSL vs. DSI Studio; MMSE correlation *R*^2^ = 97.28%, *p* < 0.001) and ROI definition method (automated vs. manual; MoCA correlation *R*^2^ = 99.99%, *p* = 0.0011) explained most of the variance.

Beyond simple correlations, mediation analyses position the DTI-ALPS index as a pivotal link within the AD pathological cascade. Multiple studies have identified the DTI-ALPS index as a significant mediator in the pathway from Aβ and tau burden to cognitive impairment (Hsu et al., 2023; Hong et al., 2024). However, the precise directionality of this mediation remains debated. Some models suggest that Aβ and tau pathology drive cognitive decline by first impairing GS function (Hsu et al., 2023). In contrast, other causal and longitudinal analyses propose an alternative sequence in which GS dysfunction acts as an upstream driver that precedes and accelerates Aβ accumulation, which in turn leads to cognitive decline (Huang et al., 2024; Zhang Y. et al., 2025). This complexity is further highlighted by findings that the mediating role of the DTI-ALPS index can itself be modulated by factors such as gray matter integrity (Chang et al., 2023).

The DTI-ALPS index also possesses independent predictive value for clinical progression, offering unique information beyond established biomarkers. Longitudinal studies have demonstrated that a lower baseline DTI-ALPS index is a significant independent predictor of future cognitive decline (Huang et al., 2024; Jungwon et al., 2025). Its inclusion in diagnostic models has been shown to significantly improve the prediction of late-stage cognitive impairment (You et al., 2025b). This independent predictive power is further underscored by genetic evidence from Mendelian randomization studies, which established that a genetically determined reduction in the DTI-ALPS index is a causal risk factor for AD (Wu et al., in press).

### 3.4 The role of the DTI-ALPS index in the pathological cascade of AD

#### 3.4.1 Progressive GS dysfunction across the AD spectrum

Cross-sectional studies have consistently demonstrated a stepwise decline in the DTI-ALPS index with increasing AD severity. This trajectory was initially characterized in early validation cohorts, where the DTI-ALPS index was found to be significantly lower across all patient groups (including AD, MCI, and VCI) compared to cognitively normal controls (Liang et al., 2023). This trend has been corroborated across cohorts from the ADNI, UK Biobank, and Asian populations (including Chinese and Korean cohorts; Huang et al., 2024; Guo et al., 2025; Xia et al., 2025; You et al., 2025b). This progressive reduction from cognitively normal (CN) through SCD and MCI to AD dementia supports GS dysfunction as a core pathophysiological process that intensifies alongside neurodegeneration (Hong et al., 2024; Schirge et al., 2025).

**Figure 4:**
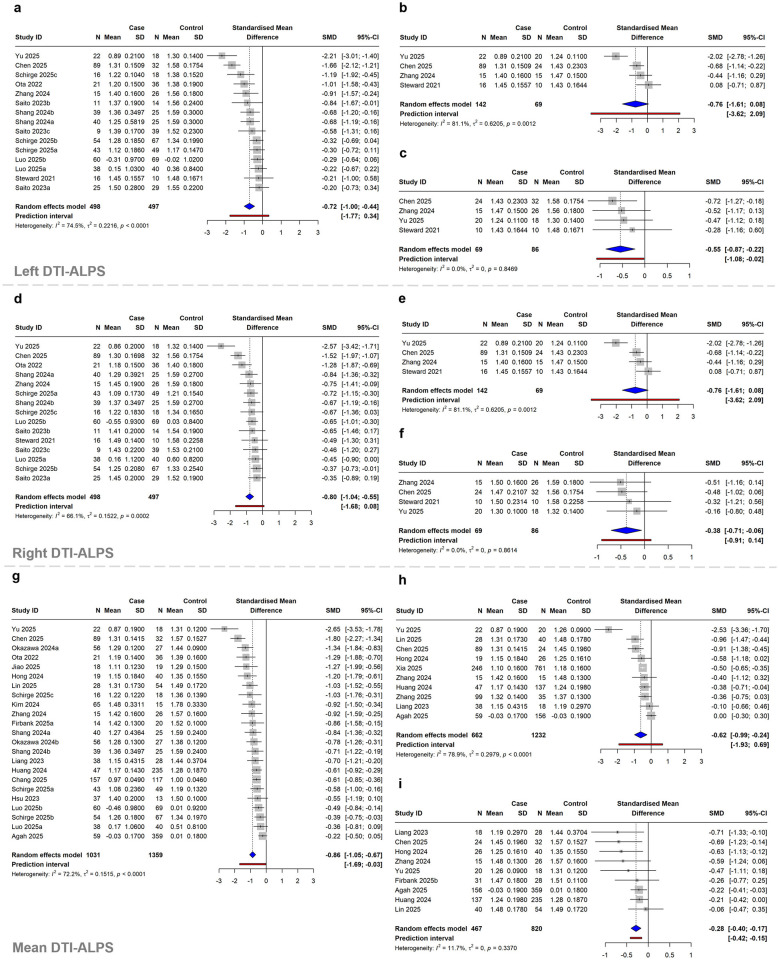
Forest plots illustrating differences in the DTI-ALPS index across diagnostic subgroups. **(a–c)** Comparisons of the left DTI-ALPS index between AD and CN **(a)**, AD and MCI **(b)**, and MCI and CN **(c)** groups. **(d–f)** Comparisons of the right DTI-ALPS index between AD and CN **(d)**, AD and MCI **(e)**, and MCI and CN **(f)** groups. **(g–i)** Comparisons of the mean DTI-ALPS index between AD and CN **(g)**, AD and MCI **(h)**, and MCI and CN **(i)** groups.

Figure 4 presents the effect sizes for differences in DTI-ALPS means across diagnostic subgroups. Notably, with the exception of the left and right DTI-ALPS index between the AD and MCI groups, all other inter-group comparisons yielded statistically significant standardized mean differences. The pooled mean differences in the DTI-ALPS index remained significant only for the AD vs. CN and MCIvs. CN comparisons after trim-and-fill adjustment (Table 2; Supplementary Figure 5). Meta-regression analysis revealed that differences in the standard deviation of age (*R*^2^ = 100.00%, *p* = 0.0010), DTI fitting software (*R*^2^ = 100.00%, *p* = 0.0006) and the proportion of females (*R*^2^ = 75.11%, *p* = 0.01) between groups significantly explained the heterogeneity solely in the comparison of the left DTI-ALPS index between the AD and MCI groups. Similarly, heterogeneity specific to the comparisons of left and right DTI-ALPS index between AD and CN groups was significantly explained by study cohort source (public datasets vs. clinical datasets; Left: *R*^2^ = 66.70%, *p* = 0.0004; Right: *R*^2^ = 76.31%, *p* = 0.0003). No moderators capable of explaining the observed heterogeneity were identified in the remaining comparisons.

A key finding is that GS impairment appears to occur early in the AD continuum. Evidence suggests that GS dysfunction occurs at the earliest stages of AD. For example, Li et al. (2024) reported in a large study that the DTI-ALPS index levels were significantly lower in SCD patients than in healthy controls and that its discriminative power for SCD patients surpassed that of conventional DTI metrics. Another PET/MR study similarly reported significantly reduced the DTI-ALPS index even at the preclinical AD stage, suggesting that GS impairment may precede overt cerebral hypoperfusion, representing an early event in the AD pathological cascade (Jiao et al., 2025). The results of the meta-analysis further corroborate this characteristic, revealing significant differences in the means and distributions of the DTI-ALPS index between the MCI and CN groups. Conversely, no significant differences were observed between the AD and MCI groups following the trim-and-fill analysis.

#### 3.4.2 Temporal positioning of GS dysfunction in the AD cascade

The precise timing of GS dysfunction within the AD cascade (A/T/N) remains a subject of complex debate, with evidence supporting both upstream and downstream roles.

Compelling evidence suggests GS dysfunction precedes overt pathology. Mendelian randomization studies indicate that genetically determined low DTI-ALPS index causally increase AD risk (Wu et al., in press). Longitudinally, baseline DTI-ALPS abnormalities appear before changes in CSF Aβ42 levels (Huang et al., 2024), and the rate of DTI-ALPS decline tracks more closely with Aβ accumulation than with atrophy (Okazawa et al., 2024), suggesting that fluid stagnation acts as a driver accelerating protein deposition.

Conversely, substantial data position DTI-ALPS decline as a secondary event coupled with neurodegeneration. DTI-ALPS index often correlate robustly with gray matter atrophy and cognitive decline but lack a direct link to cerebral Aβ burden (Kim et al., 2024). Some cohorts show significant DTI-ALPS reduction only during the dementia stage rather than MCI (Yu et al., 2025), implying it may reflect structural damage following advanced pathology (Hsu et al., 2023).

This apparent contradiction likely stems from disease heterogeneity and methodological confounding. The relationship varies by subtype. For instance, the DTI-ALPS index links to Aβ in early-onset AD but to vascular factors in late-onset AD (Luo et al., 2025). Furthermore, because the DTI-ALPS signal relies on white matter integrity, observed changes likely represent a composite of early fluid dynamic failure and later structural degeneration (Li et al., 2025).

#### 3.4.3 Longitudinal predictive value of the DTI-ALPS index in AD progression

Longitudinal studies further substantiate the potential of the DTI-ALPS index as an important biomarker for predicting AD disease progression. A body of research robustly demonstrates that the baseline DTI-ALPS index can effectively predict future cognitive trajectories and clinical state transitions. For example, lower baseline DTI-ALPS index is significantly associated with faster rates of Aβ deposition, brain atrophy, and cognitive decline (Huang et al., 2024). In SCD and CN populations, lower DTI-ALPS index also predict a greater risk of cognitive worsening (Li et al., 2024). Among MCI patients, those with lower baseline DTI-ALPS index have a significantly increased risk of conversion to AD dementia and are associated with faster cognitive decline over subsequent years (Guo et al., 2025). A study by Bao et al. (2025) even quantified this protective effect, indicating that a well-functioning GS could delay the onset of MCI and AD by approximately 3.5 years.

Despite compelling evidence, the predictive power of the DTI-ALPS index is not absolute and may be influenced by disease stage. A 1-year longitudinal study revealed that in patients with established AD, baseline tau burden was a stronger predictor of cognitive decline than the DTI-ALPS index (Chang et al., 2025). This suggests that the relative predictive importance of different biomarkers evolve across the disease continuum that GS function is a key predictor in the early-to-mid stages, while in the presence of advanced tau pathology, its influence is superseded by the associated neurodegenerative changes as the primary driver of cognitive decline. Therefore, integrating the DTI-ALPS index into multimodal biomarker models is pivotal for achieving precise prognostic predictions in future research.

### 3.5 Methodological advancements and standardization

Recent research has focused on enhancing the reproducibility, biological specificity, and interpretative validity of the DTI-ALPS method.

To address the operator variability inherent in early manual ROI delineation (Taoka et al., 2017), recent studies have established standardized selection strategies (Steward et al., 2021) and fully automated, open-source computational pipelines (Liu et al., 2024). These advancements ensure high reliability across large-scale datasets. Furthermore, to mitigate scanner-induced heterogeneity in multicenter studies, the ComBat harmonization technique (Saito et al., 2023a) and direction-preserving algorithms (vALPS; Saito et al., 2023b) have been introduced. Optimization of acquisition parameters, specifically using b = 1,000 s/mm^2^, has also been confirmed to yield more stable results (Taoka et al., 2017; Okazawa et al., 2024).

A critical challenge lies in ensuring the DTI-ALPS index reflects glymphatic function rather than coexisting structural pathologies. To decouple glymphatic dysfunction from cerebral small vessel disease, Kim et al. (2024) incorporated WMH as a covariate in statistical models. More fundamentally, Li et al. (2025) developed a “corrected DTI-ALPS index” validated against postmortem tissue to isolate fluid dynamics from age- and disease-related white matter microstructural confounds.

As biomarker models become more complex, the unique predictive value of the DTI-ALPS index has been scrutinized. Bayesian model averaging revealed that its independent explanatory power for cognitive decline is limited when analyzed alongside other white matter integrity markers (e.g., FW), indicating high multicollinearity (Agah et al., 2025). This underscores the necessity of interpreting the DTI-ALPS index cautiously as part of a synergistic biomarker panel rather than in isolation.

### 3.6 The DTI-ALPS index as a converging point of multiple pathophysiological pathways

The DTI-ALPS index serves as a pivotal hub within the AD pathological network, integrating upstream vascular and systemic risks with downstream neurodegeneration.

Glymphatic function is fundamentally driven by cerebral arterial pulsations. Reduced DTI-ALPS index correlate with arterial stiffness (You et al., 2025a) and systemic vascular risks like ischemic heart disease (Wang et al., 2025), forming a “vascular–glymphatic axis.” This convergence extends to physiological states, sleep disturbances (Shang et al., 2024) and retinal vascular changes (Chen Z. et al., 2025) have been shown to impair cognition specifically through the mediation of GS dysfunction.

Mechanistically, the DTI-ALPS index mediates the impact of core pathologies on neuronal function. Jiao et al. (2025) revealed a sequential pathway where Aβ burden leads to vascular deficits, which in turn impair glymphatic clearance, ultimately causing cognitive decline. Crucially, the DTI-ALPS index provides unique prognostic value independent of traditional cerebral small vessel disease markers (Kim et al., 2024; You et al., 2025b), suggesting it captures a distinct pathophysiological process beyond structural vascular damage.

At the cellular level, macroscopic fluid stagnation linked to lower DTI-ALPS index correlates with elevated plasma markers of neuronal injury (NfL) and astrogliosis (GFAP; Firbank et al., 2025). However, this relationship is complex; in cases of primary tau-driven hippocampal atrophy, the mediating role of glymphatic dysfunction may be less prominent compared to astrocytic activation (Chang et al., 2025), highlighting the need to consider disease subtypes.

## 4 Discussion

This systematic review examines the application of the DTI-ALPS index in AD research. As a non-invasive metric of brain GS function, the DTI-ALPS index has been widely employed in AD studies. Synthesizing the findings from the included studies, we identify a robust association between the DTI-ALPS index and neurodegenerative changes (N) as well as cognitive decline in AD pathology (Kim et al., 2024; Firbank et al., 2025; Li et al., 2025). Several moderators, including covariate adjustment, female ratio, DTI fitting software, and PET tracer type, were associated with heterogeneity, suggesting that both methodological and cohort-composition factors influence the observed DTI-ALPS–Aβ relationship. Consequently, the direct association between the DTI-ALPS index and Aβ and tau pathologies remains controversial, particularly when controlling for confounders such as white matter microstructural integrity, raising questions about the specificity of such associations (Li et al., 2025). This ambiguity extends to the precise temporal positioning of GS dysfunction as captured by the DTI-ALPS index within the AD pathological cascade (Hsu et al., 2023; Huang et al., 2024; Wu et al., in press), indicating that its independent biomarker value requires further validation.

Therefore, although the DTI-ALPS index measure has been extensively utilized in AD research and holds considerable promise for elucidating AD pathophysiology, its translation from a research tool to clinical practice remains challenging. In this section, we focus on potential reasons for the conflicting findings regarding the correlation and temporal sequence between the DTI-ALPS index and A/T pathologies, the role of the DTI-ALPS index in AD pathology and its modulating factors, methodological limitations of the DTI-ALPS approach, and future research directions. Our aim is to clarify the sources of current discrepancies and help steer subsequent investigations in this field.

### 4.1 Methodological and biological complexity: interpreting contradictions within the A/T/N framework

**Figure 5:**
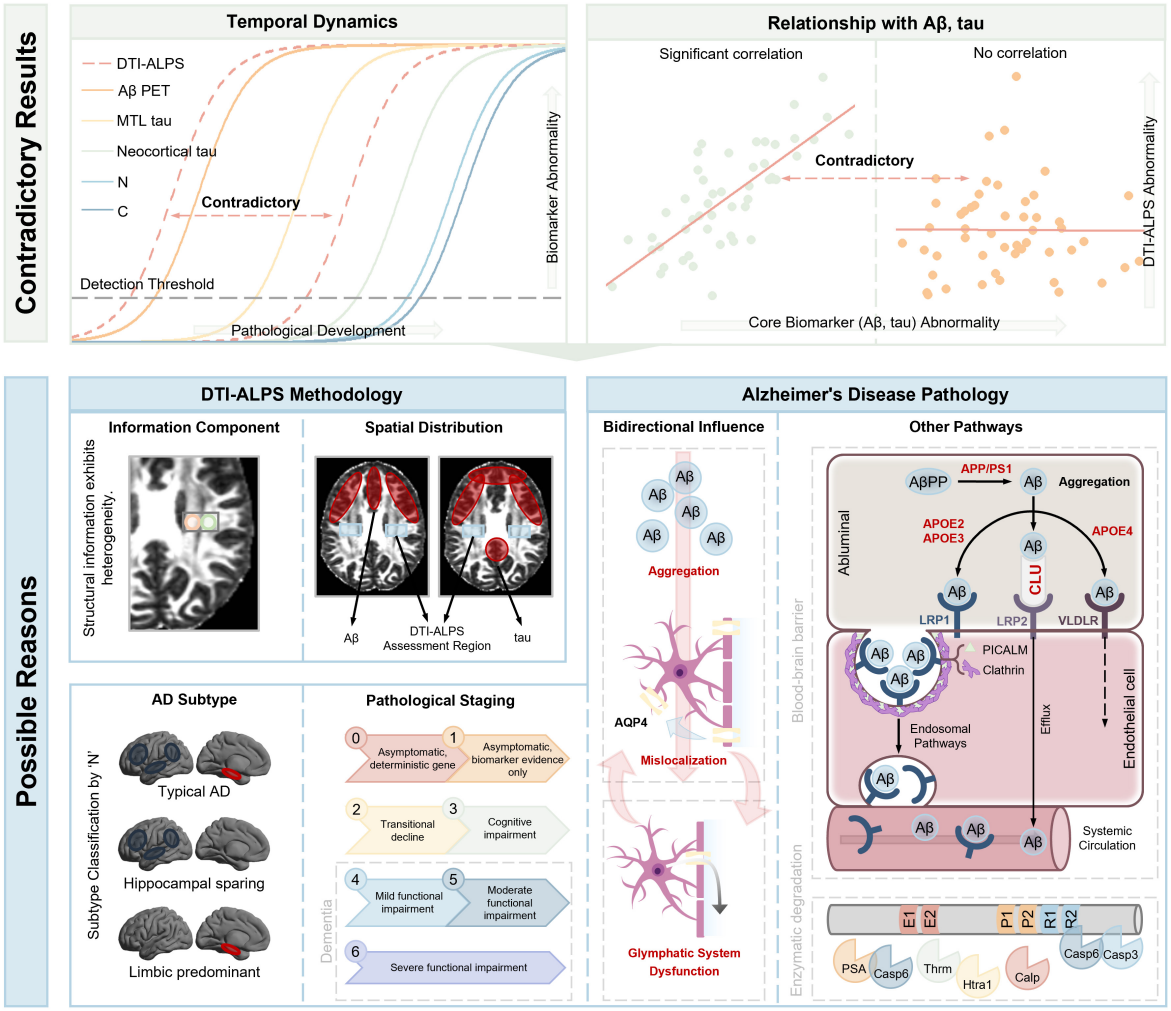
Controversies in the main findings of the DTI-ALPS index in AD research and possible reasons.

The divergent findings regarding the DTI-ALPS index within the A/T/N framework for AD primarily revolve around its associations with Aβ and tau pathology, as well as the temporal positioning of the DTI-ALPS index within this framework. Rather than being mutually exclusive, these conflicting results may stem from similar underlying factors, namely the inherent methodological limitations of the DTI-ALPS index and the complexity of AD pathophysiology, as depicted in Figure 5.

First, methodological variability and signal non-specificity significantly contribute to inconsistent results. The DTI-ALPS index relies on diffusion anisotropy to infer fluid movement (Taoka, 2021), so it represents a composite signal incorporating significant contributions from white matter microstructure, lesions, and atrophy (Naganawa et al., 2018; Taoka, 2021; Siow et al., 2022; Song et al., 2022; Hsu et al., 2023). Notably, evidence shows that the DTI-ALPS-Aβ association disappears when correcting for these structural components (Li et al., 2025). This biological ambiguity is exacerbated by operator variability. Inconsistencies in ROI placement and size alter the proportion of tissue types analyzed (Taoka et al., 2017; Saito et al., 2023a; Zhang et al., 2024), as protocols vary widely from small spheres or voxels (Steward et al., 2021; Jungwon et al., 2025) to larger regions (Taoka et al., 2017; Hong et al., 2024; Huang et al., 2024; Chen F. et al., 2025; Luo et al., 2025; Zhang Q. et al., 2025). This explains why the DTI-ALPS index acts as an integrative marker correlating logically with global cognition while showing unstable associations with specific proteins. Furthermore, its unique predictive power diminishes in multivariate models because of multicollinearity (Agah et al., 2025). Another key consideration involves the spatial mismatch between localized DTI-ALPS measurements and distributed AD pathology. The DTI-ALPS index is computed solely at the lateral ventricles (Taoka et al., 2017) with the aim of representing whole-brain function. However, AD pathology is inherently regional, as early Aβ deposition targets specific networks such as the default mode network and precuneus (Cho et al., 2016; Grothe et al., 2017; Palmqvist et al., 2017; Lowe et al., 2019; Mattsson et al., 2019). Similarly, Tau spreads systematically from medial temporal areas to association cortices (Braak and Braak, 1991). This anatomical discordance between the measurement site and pathological loci likely attenuates direct correlations and complicates longitudinal tracking. These limitations underscore the need for future development of region-specific glymphatic assessment methods.

Sex composition may also explain part of the between-study heterogeneity. In our meta-regression, the study-level female ratio was associated with heterogeneity in the DTI-ALPS–Aβ PET relationship, and between-group differences in female proportion also contributed to heterogeneity in the left DTI-ALPS comparison between AD and MCI. This finding should be interpreted cautiously, because female ratio is a study-level variable and may reflect correlated factors such as age, intracranial volume, APOE ε4 distribution, vascular burden, menopausal status, and recruitment strategy. Still, the result is biologically plausible: DTI-ALPS is partly shaped by brain morphometry, and sex may influence the observed DTI-ALPS–AD biomarker association through menopause-related endocrine changes, sleep–glymphatic interactions, and faster tau accumulation in women with elevated Aβ burden or APOE ε4 carrier status (Ekanayake et al., 2024; Ozsahin et al., 2024; Coughlan et al., 2025; Satpathi et al., 2025). Thus, the role of female ratio in our analysis is unlikely to represent a simple sex effect; it more likely reflects the combined influence of sex-related morphometric differences and sex-related vulnerability to AD pathology.

Second, the complexity of AD pathophysiology extends beyond simple linear causal models (Jack et al., 2024), with heterogeneity serving as a critical confounder. The association between the DTI-ALPS index and A/T pathology is likely subtype-dependent rather than uniform. AD manifests as distinct subtypes with heterogeneous spreading patterns (e.g., limbic-predominant, hippocampal-sparing, or typical AD; Gosche et al., 2002; Jack et al., 2002; Csernansky et al., 2004; Whitwell et al., 2008, 2012; Vogel et al., 2021; Collij et al., 2022). In limbic-predominant subtypes, where tau pathology originates in the medial temporal lobe near the lateral ventricles, fiber tract integrity in the DTI-ALPS measurement region may be affected early, leading to strong correlations (Meyer-Luehmann et al., 2008; Chen et al., 2017; Liu et al., 2020). Conversely, in hippocampal-sparing or cortical-predominant subtypes, initial pathology is spatially distant from the ventricles. In these cases, DTI-ALPS index alterations may only appear later as a downstream consequence of widespread diffuse axonal injury and neuroinflammation (Clavaguera et al., 2015; Novoa et al., 2022). This spatial mismatch implies that the timing and strength of the DTI-ALPS-pathology association vary fundamentally across patients. Furthermore, reliance on broad clinical categories (CN, MCI, AD) rather than fine-grained biological staging (A/T/N framework) masks underlying variation. Current literature largely overlooks that pathological dynamics differ significantly across granular biomarker stages (e.g., from A+T2– to A+T2HIGH+; Tetzloff et al., 2018; Knopman et al., 2021; Sanchez et al., 2021; Vogel et al., 2021; Leuzy et al., 2022; Jack et al., 2024). Pooling participants with vastly different underlying pathological states into coarse groups likely obscures the true temporal relationship between glymphatic integrity and disease progression.

Finally, two additional confounding factors complicate these findings. One is the interaction between GS dysfunction and AD pathology is bidirectional rather than linear. Pathological progression itself disrupts clearance mechanisms, as evidenced by the association between AQP4 mislocalization and increased Aβ burden (Zeppenfeld et al., 2017). Conversely, GS dysfunction accelerates solute accumulation (Mohaupt et al., 2023) and creates a vicious cycle of pathology. Another factor is the GS operates alongside other clearance systems such as trans-blood-brain barrier transport (Shibata et al., 2000; Deane et al., 2003; Zlokovic, 2011; Ma et al., 2018) and enzymatic degradation (Iwata et al., 2001; Farris et al., 2003; Saido and Leissring, 2012). The brain may compensatorily upregulate these alternative pathways when GS function is impaired (Tarasoff-Conway et al., 2015; Nelson et al., 2016). However, the efficacy of this compensation exhibits considerable individual variation. This variability likely obscures the observed relationship between the DTI-ALPS index and pathological outcomes, as patients with similar DTI-ALPS scores may have vastly different total clearance capacities depending on their compensatory reserves.

### 4.2 The DTI-ALPS index as a hub converging multiple pathological pathways

Although studies on AD based on the DTI-ALPS index have yielded inconsistent findings, this may precisely reflect the pivotal role of the GS as a key hub within the AD pathological network. The traditional neurovascular hypothesis has established the cerebral vasculature as a convergence point for damage induced by genetic, environmental, and lifestyle risk factors, and posits that primary vascular injury is a core driver accelerating AD pathology (Zlokovic, 2011; Sweeney et al., 2015). However, this hypothesis has not fully elucidated the mechanisms underlying the clearance of pathogenic proteins from the brain parenchyma. The recently discovered GS provides a critical complement by revealing a direct link between cerebrovascular health and protein homeostasis. We propose that GS dysfunction acts as a central mediator, linking upstream risk factors to downstream neurodegeneration. This perspective does not contradict the neurovascular hypothesis but rather refines it. Specifically, healthy cerebral vascular pulsations serve as a primary driving force for GS fluid flow (You et al., 2025a). Therefore, systemic vascular risk factors can impair GS clearance efficiency by weakening this driving force (Chen Z. et al., 2025; Wang et al., 2025). A compromised GS leads to impaired clearance of Aβ and hyperphosphorylated Tau. The accumulation of these pathological proteins, in turn, exacerbates GS dysfunction by inducing reactive astrogliosis and abnormal polarization of AQP4, thereby forming a vicious cycle (Zeppenfeld et al., 2017; Mohaupt et al., 2023). The resulting neuroinflammation, neuronal damage, and neurofibrillary tangles (Firbank et al., 2025) ultimately culminate in cognitive decline.

**Figure 6:**
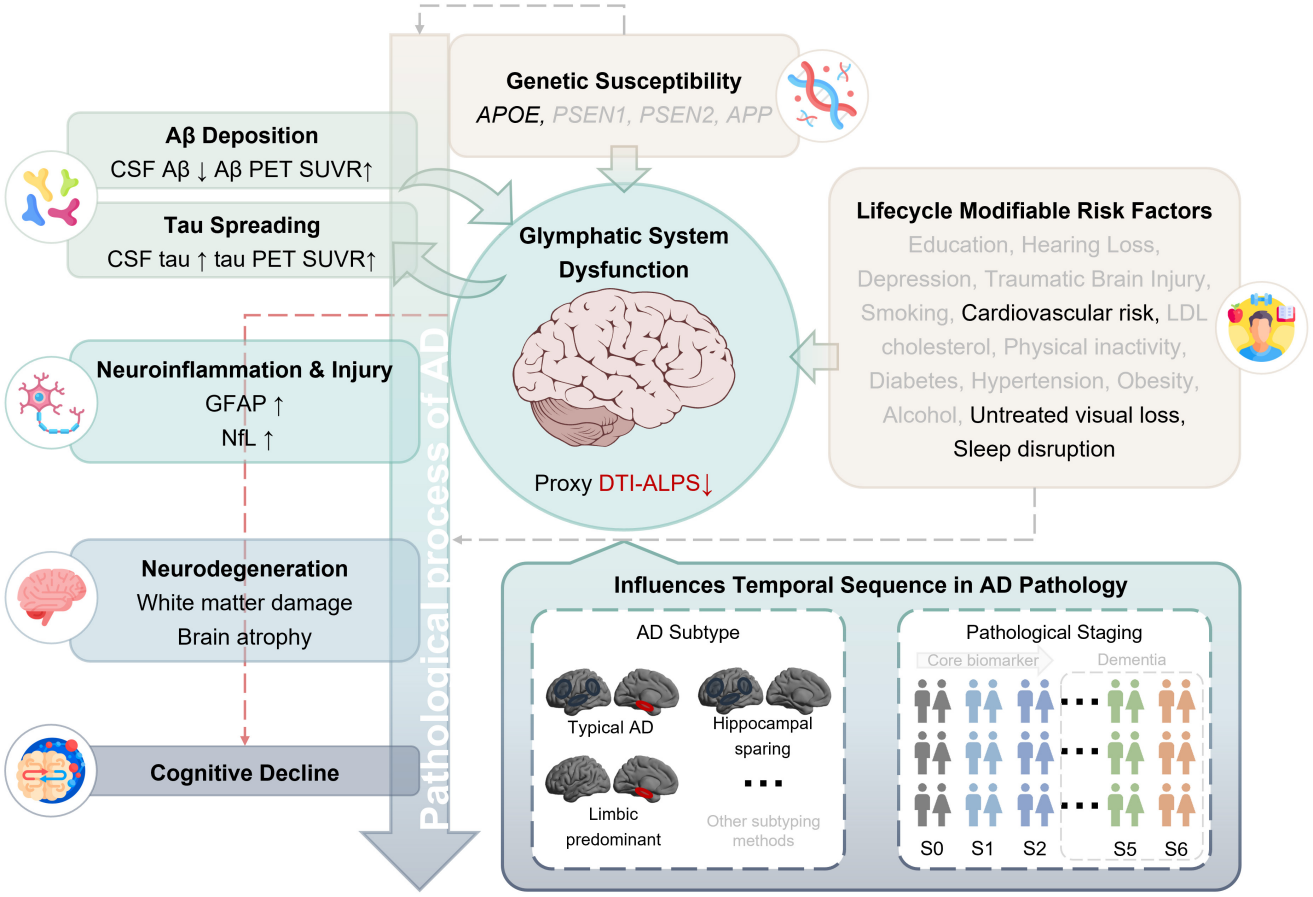
An integrative framework for the role of the DTI-ALPS index in AD pathogenesis.

Synthesizing these findings, we propose the “GS Hub Integrative Framework.” As illustrated in Figure 6, the core tenet of this model is that various known AD risk factors (such as vascular health, genotype, lifestyle, and environmental exposures) converge to impair GS function through a common pathway. This disruption of cerebral protein clearance homeostasis subsequently triggers or accelerates the neurodegenerative cascade in AD. Although several pathways have gained qualitative or quantitative support, we emphasize that the framework currently constitutes a plausible inferential hypothesis built upon cross-sectional studies and a limited number of longitudinal investigations, and the precise mechanisms of each pathway await elucidation through more rigorous experimental studies. Upstream, vascular risk factors are closely associated with the DTI-ALPS index: evidence indicates that the DTI-ALPS index shows strong correlations with arterial stiffness (You et al., 2025a) and systemic vascular risks such as ischemic heart disease (Wang et al., 2025), while associations with WMH, enlarged PVS, and choroid plexus volume (Hong et al., 2024; Sacchi et al., 2024; Zhang et al., 2024; Xia et al., 2025) provide indirect support. Downstream, the stable relationships between glymphatic function indexed by DTI-ALPS and Aβ deposition (Section 3.3.1) as well as cognitive scores (Section 3.3.3) link glymphatic dysfunction to the AD pathological spectrum. Thus, the DTI-ALPS index is not merely an imaging tool for assessing GS function but may also serve as a quantifiable window into early pathological events preceding macroscopic structural atrophy. This offers a novel theoretical foundation and potential targets for risk assessment and intervention in AD.

### 4.3 Limitations and future directions of the DTI-ALPS method

Despite its promise as a non-invasive biomarker for GS function and AD pathophysiology, translating the DTI-ALPS index into clinical practice faces challenges requiring methodological standardization and biological validation.

#### 4.3.1 Methodological standardization and biological validation

Methodological limitations primarily drive inter-study heterogeneity. Current reliance on manual ROI delineation introduces operator variability, compromising reproducibility (Taoka et al., 2017; Saito et al., 2023a). The differences in the preprocessing pipeline of dMRI and the fitting method of DTI also significantly affect the robustness of the results. Future research must adopt standardized, fully automated pipelines, such as recently developed open-source tools (Liu et al., 2024), to ensure reliability across multi-center datasets.

Additionally, the DTI-ALPS index represents a composite signal influenced by white matter microstructure and atrophy rather than a pure measure of GS activity. Although correction algorithms exist, they lack sufficient postmortem validation (Li et al., 2025). Future studies must critically account for these structural confounds.

Furthermore, the localized measurement of the DTI-ALPS index near lateral ventricles may be spatially discordant with heterogeneous AD pathologies like Aβ and tau (Vogel et al., 2021; Collij et al., 2022). Developing region-specific DTI-ALPS methodologies is essential to capture whole-brain or network-specific GS function. Finally, as an indirect proxy, the DTI-ALPS index requires cross-validation with direct measures, such as intrathecal contrast-enhanced MRI (Eide and Ringstad, 2015), to quantify its relationship with actual fluid exchange efficiency.

Finally, the DTI-ALPS index should not be interpreted in isolation but rather alongside several related diffusion-derived metrics. The FW model decomposes diffusion into tissue and free-water components, the FW fraction exhibits multicollinearity with the DTI-ALPS index (Agah et al., 2025), reflecting shared fluid-related signals, and therefore should not be entered into the same predictive model without collinearity diagnostics. Conventional DTI metrics (MD, FA) covary with white matter microstructural integrity and may confound the interpretation of DTI-ALPS in regions with marked axonal pathology (Naganawa et al., 2018; Siow et al., 2022; Song et al., 2022; Li et al., 2025). NODDI-derived metrics capture intra-axonal compartments and complement the DTI-ALPS index, which is oriented toward perivascular fluid dynamics (Palacios et al., 2020). Diffusion acquisition schemes and IVIM-related perfusion indices can further characterize the coupling between glymphatic flow and microcirculation (Liu et al., 2022), yielding information not accessible from DTI-ALPS alone. Given the inherent limitations of each metric, the most informative interpretation is to treat the DTI-ALPS index as one component within a panel of diffusion metrics and to explicitly model their shared variance. We therefore recommend that future studies report the DTI-ALPS index alongside at least one neurodegenerative microstructural metric and quantify the unique contribution of each.

#### 4.3.2 Deepening clinical and pathophysiological research

Research must evolve from descriptive observations to mechanistic exploration and clinical translation. Although closely associated with clinical progression, the utility of the DTI-ALPS index as an endpoint in interventional trials targeting GS function [e.g., sleep modulation (Shang et al., 2024), physical exercise, or AQP4-targeted therapeutics] remains largely unexplored.

Moreover, AD heterogeneity contributes to inconsistent findings. Previous studies often relied on coarse clinical staging. Future research should implement refined, biomarker-based stratification [e.g., A/T/N pathological subtypes (Whitwell et al., 2012; Vogel et al., 2021)] to elucidate DTI-ALPS alterations across specific AD subtypes and validate its potential as a diagnostic or prognostic biomarker for distinct subpopulations.

The DTI-ALPS index lacks disease specificity, as declines have been observed in Parkinson's disease, dementia with Lewy bodies, vascular cognitive impairment, and normal aging, limiting its standalone diagnostic utility. Moreover, its incremental value over Aβ PET, CSF Aβ42/40, and plasma p-tau217 has not been confirmed by direct comparative studies within the same cohort. Nevertheless, within the AD continuum, the index has at least four rational complementary applications: In the SCD or CN stage, DTI-ALPS alterations may emerge before overt Aβ PET positivity (Section 3.4), suggesting potential for identifying high-risk individuals while established biomarkers remain negative; In cases with substantial coexisting cerebral small vessel disease burden, the index can capture contributions along the vascular–glymphatic axis that Aβ PET and plasma p-tau217 may underestimate (Hong et al., 2024; Sacchi et al., 2024); For longitudinal monitoring of glymphatic-targeted interventions such as sleep modulation, structured physical exercise, and AQP4-targeted therapy, DTI-ALPS offers a noninvasive response indicator that can compensate for the limited detectability of short-term changes in Aβ PET and plasma p-tau217; And when entered into multivariable models alongside established biomarkers, DTI-ALPS has shown incremental predictive value (You et al., 2025b), consistent with its role as an upstream marker. Therefore, at the current stage, the DTI-ALPS index should not be used alone for diagnosis or differential diagnosis. Conducting direct comparative studies to validate its incremental value is the priority next step toward clinical translation.

### 4.4 Limitations

Several limitations warrant consideration. First, although several moderators were identified, these meta-regression results should be interpreted cautiously because many analyses included few studies and may be prone to overfitting.

Second, publication bias maybe affects robustness. Notably, the DTI-ALPS-tau correlation lost significance after adjustment. The omission of statistical data for negative findings (Kim et al., 2024; Firbank et al., 2025; Schirge et al., 2025) may introduce bias.

Third, although nine of the 36 included studies employed a longitudinal design, the majority (27/36) remained cross-sectional. The causal direction between DTI-ALPS and AD progression therefore cannot be firmly established.

Finally, excluding non-English publications may overlook relevant data. More critically, most studies relied on broad clinical categories (e.g., MCI, AD) rather than fine-grained biomarker-based stratification (A/T/N framework), preventing precise exploration of glymphatic dynamics across specific pathological stages.

## 5 Conclusion

Our systematic review confirms robust associations between the DTI-ALPS index, neurodegeneration, and cognitive decline. However, correlations with Aβ and tau pathologies remain heterogeneous, primarily due to methodological variability and biological complexity. Consequently, we posit that the DTI-ALPS index functions as a composite metric reflecting both fluid dynamics and structural integrity. Based on this, we propose the “GS Hub Integrative Framework,” postulating that diverse risk factors converge to impair the glymphatic system, thereby accelerating disease progression. To bridge the gap to clinical application, future research must prioritize methodological rigor by moving toward standardized, automated pipelines. Furthermore, validating biological specificity against direct physiological measures and implementing biomarker-based patient stratification are essential steps to establish the DTI-ALPS index as a reliable complementary imaging marker for risk stratification and therapeutic monitoring.

## Data Availability

The original contributions presented in the study are included in the article/Supplementary material, further inquiries can be directed to the corresponding authors.
