# Supplementary material for: Glymphatic dysfunction in Alzheimer's disease: a systematic review and meta-analysis of the DTI-ALPS index as an imaging biomarker
Source: Front Aging Neurosci. 2026 Jul 2;18:1832525. doi: 10.3389/fnagi.2026.1832525 (PMC13372759; doi:10.3389/fnagi.2026.1832525)
Supplement: Supplementary file 1 [file Data_Sheet_1.pdf]

Supplementary Material

1 Glymphatic System and the DTI-ALPS index in human brain

1.1 The Glymphatic System in the Human Brain

The discovery of the GS represents a pivotal advance in neuroscience, fundamentally altering our understanding of waste clearance and fluid dynamics within the CNS(Iliff et al., 2012). This system plays a critical role in transporting nutrients and neuroactive substances while removing endogenous and exogenous metabolites(Gouveia-Freitas and Bastos-Leite, 2021; Ding et al., 2023). GS circulation proceeds through three sequential stages(Natale et al., 2021; Szlufik et al., 2024): the production and bulk flow of CSF(Hablitz and Nedergaard, 2021), fluid exchange within PVS(Mestre et al., 2017), and the drainage of waste-laden interstitial fluid from the CNS(Aspelund et al., 2015).

The foundation of the GS lies in the production and flow of CSF(Lehtinen et al., 2013), which originates primarily from neuroepithelial cells in the choroid plexus. From the lateral ventricles, CSF flows through the third ventricle and the aqueduct of Sylvius to the fourth ventricle(Hladky and Barrand, 2014), eventually draining into the cisterna magna through the foramina of Magendie and Luschka. At this critical junction, CSF is either reabsorbed by arachnoid granulations or redirected into the subarachnoid space. Subsequently, CSF flows along the cerebral surface vasculature, finally influxing into the PVS of penetrating arteries that dive into the brain parenchyma.

The PVS serves as the structural framework of the GS, acting as a conduit for fluid transport(Wardlaw et al., 2020; Sepehrband et al., 2021; Tian et al., 2022). As pial arteries penetrate the parenchyma to become penetrating arteries, the pia mater invaginates to create the PVS between the vessel wall and brain tissue. CSF within the arterial PVS enters the parenchyma, mediated by aquaporin-4 (AQP4) channels highly expressed on astrocytic end-feet(Benveniste et al., 2019b), mixing with ISF within the neuropil (Figure 2). This convective flow is primarily propelled by arterial pulsation (Iliff et al., 2012; Bedussi et al., 2018) and is significantly modulated by sleep(Xie et al., 2013; Benveniste et al., 2019a; Fultz et al., 2019; Reddy and van der Werf, 2020; Licastro et al., 2024), respiration(Ozturk et al., n.d.), and body posture. Within the extracellular space, CSF and ISF integrate into a single fluid phase, enabling the exchange of inorganic ions, neurotransmitters, and extracellular matrix macromolecules(Syková and Nicholson, 2008). The CSF-ISF mixture fluid (carrying soluble amyloid-β (Aβ)(Iliff et al., 2012), tau, lactate(Lundgaard et al., 2017), and apolipoprotein E(Achariyar et al., 2016) etc.) subsequently drains into the perivenous PVS.

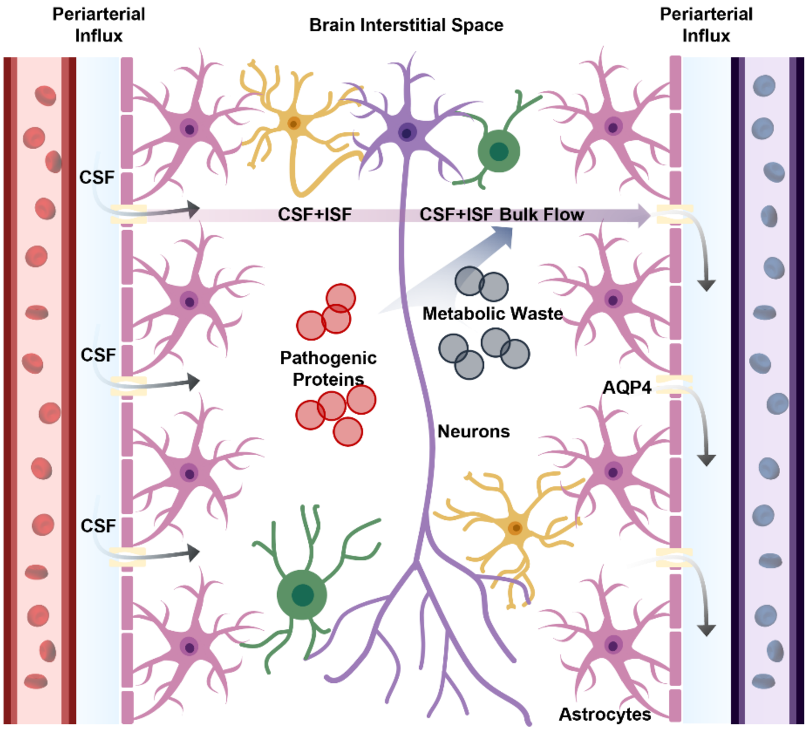

Supplementary Figure 1. Schematic diagram of dynamic fluid exchange in the GS of the brain

Meningeal lymphatic vessels provide the vital link between the brain and the peripheral lymphatic system(Aspelund et al., 2015). The CSF-ISF mixture egresses along the perivenous PVS into meningeal lymphatics(Formolo et al., 2023), ultimately draining into deep cervical lymph nodes. This process completes the clearance circuit from the CNS to the periphery.

Through these three sequential stages, the GS efficiently facilitates cerebral waste clearance and maintains microenvironmental homeostasis. The integrity of this pathway is indispensable for neurological function. Consequently, GS dysfunction has been identified as a central pathological component in various neurodegenerative disorders. Particularly in Alzheimer's and Parkinson's diseases, impaired GS function directly contributes to the accumulation and aggregation of neurotoxic proteins, including Aβ, tau, and α-synuclein(Silva et al., 2021; Buccellato et al., 2022). As such, GS function is increasingly recognized as a potential biomarker for monitoring neurodegenerative disease progression.

1.2 Principle of the DTI-ALPS index

The DTI-ALPS index is a noninvasive method designed to evaluate GS activity in the human brain. This technique was originally introduced by Taoka et al. in 2017(Taoka et al., 2017). The assessment is based on the distinct spatial arrangement of medullary veins, projection fibers, and association fibers.

As illustrated in Figure 3, at the level of the lateral ventricle body, the medullary veins run perpendicular to the ventricular wall, and the PVS aligns with the medullary veins in the left–right direction (X-axis). On axial slices through this region, projection fibers are oriented in the superior-

inferior direction (Z-axis), primarily adjacent to the lateral ventricles, whereas the superior longitudinal fasciculus (a major association fiber tract) runs in the anterior–posterior direction (Y-axis), located lateral to the projection fibers. This spatial arrangement results in a unique orthogonal configuration among the PVS, projection fibers, and superior longitudinal fasciculus, allowing diffusion analysis along the PVS to be performed in a relatively independent manner.

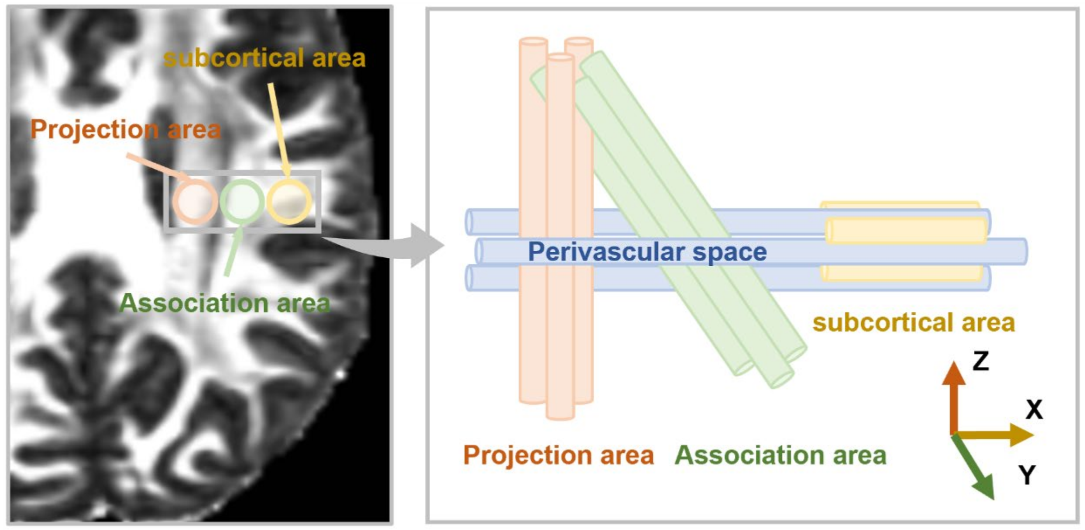

Supplementary Figure 2. Distribution of projection fibers, association fibers, subcortical fibers, and PVS

Histological alterations in the left–right direction is expected to similarly affect both projection and association fibers. Therefore, if changes are observed in both fiber systems, it is highly probable that at least some of these alterations originate from the GS. On the basis of this reasoning, Taoka et al. placed ROIs in the lateral ventricular region on DTI and evaluated diffusivity along the PVS direction at the level of the lateral ventricular body, comparing it with diffusivity in projection and association fibers to derive the DTI-ALPS index(Taoka et al., 2017).

The DTI-ALPS index is defined as the ratio of the average diffusivity along the x-axis in the projection fiber region (D_{x-proj}) and association fiber region ($D_{x-assoc}$) to the average diffusivity along the y-axis in the projection fiber region (D_{y-proj}) and the z-axis in the association fiber region ($D_{z-assoc}$). The formula is expressed as follows:

$$DTI - ALPS = \frac{mean(D_{x-proj}, D_{x-assoc})}{mean(D_{y-proj}, D_{z-assoc})} \quad (1)$$

2 References

Achariyar, T. M., Li, B., Peng, W., Verghese, P. B., Shi, Y., McConnell, E., et al. (2016). Glymphatic distribution of CSF-derived apoE into brain is isoform specific and suppressed during sleep deprivation. *Mol Neurodegener* 11, 74. doi: 10.1186/s13024-016-0138-8

Aspelund, A., Antila, S., Proulx, S. T., Karlsen, T. V., Karaman, S., Detmar, M., et al. (2015). A dural lymphatic vascular system that drains brain interstitial fluid and macromolecules. *J Exp Med* 212, 991–999. doi: 10.1084/jem.20142290

Bedussi, B., Almasian, M., de Vos, J., VanBavel, E., and Bakker, E. N. (2018). Paravascular spaces at the brain surface: Low resistance pathways for cerebrospinal fluid flow. *J Cereb Blood Flow Metab* 38, 719–726. doi: 10.1177/0271678X17737984

Benveniste, H., Heerdt, P. M., Fontes, M., Rothman, D. L., and Volkow, N. D. (2019a). Glymphatic System Function in Relation to Anesthesia and Sleep States. *Anesthesia & Analgesia* 128, 747. doi: 10.1213/ANE.0000000000004069

Benveniste, H., Liu, X., Koundal, S., Sanggaard, S., Lee, H., and Wardlaw, J. (2019b). The glymphatic system and waste clearance with brain aging. *Gerontology* 65, 106–119. doi: 10.1159/000490349

Buccellato, F. R., D'Anca, M., Serpente, M., Arighi, A., and Galimberti, D. (2022). The Role of Glymphatic System in Alzheimer's and Parkinson's Disease Pathogenesis. *Biomedicines* 10, 2261. doi: 10.3390/biomedicines10092261

Ding, Z., Fan, X., Zhang, Y., Yao, M., Wang, G., Dong, Y., et al. (2023). The glymphatic system: a new perspective on brain diseases. *Front. Aging Neurosci.* 15. doi: 10.3389/fnagi.2023.1179988

Formolo, D. A., Yu, J., Lin, K., Tsang, H. W. H., Ou, H., Kranz, G. S., et al. (2023). Leveraging the glymphatic and meningeal lymphatic systems as therapeutic strategies in Alzheimer's disease: an updated overview of nonpharmacological therapies. *Mol Neurodegener* 18, 26. doi: 10.1186/s13024-023-00618-3

Fultz, N. E., Bonmassar, G., Setsompop, K., Stickgold, R. A., Rosen, B. R., Polimeni, J. R., et al. (2019). Coupled electrophysiological, hemodynamic, and cerebrospinal fluid oscillations in human sleep. *Science* 366, 628–631. doi: 10.1126/science.aax5440

Gouveia-Freitas, K., and Bastos-Leite, A. J. (2021). Perivascular spaces and brain waste clearance systems: relevance for neurodegenerative and cerebrovascular pathology. *Neuroradiology* 63, 1581–1597. doi: 10.1007/s00234-021-02718-7

Hablitz, L. M., and Nedergaard, M. (2021). The Glymphatic System: A Novel Component of Fundamental Neurobiology. *J Neurosci* 41, 7698–7711. doi: 10.1523/JNEUROSCI.0619-21.2021

Hladky, S. B., and Barrand, M. A. (2014). Mechanisms of fluid movement into, through and out of the brain: evaluation of the evidence. *Fluids Barriers CNS* 11, 26. doi: 10.1186/2045-8118-11-26

Iliff, J. J., Wang, M., Liao, Y., Plogg, B. A., Peng, W., Gundersen, G. A., et al. (2012). A Paravascular Pathway Facilitates CSF Flow Through the Brain Parenchyma and the Clearance of Interstitial Solutes, Including Amyloid β. *Science Translational Medicine* 4, 147ra111-147ra111. doi: 10.1126/scitranslmed.3003748

Lehtinen, M. K., Bjornsson, C. S., Dymecki, S. M., Gilbertson, R. J., Holtzman, D. M., and Monuki, E. S. (2013). The Choroid Plexus and Cerebrospinal Fluid: Emerging Roles in Development,

Disease, and Therapy. *J Neurosci* 33, 17553–17559. doi: 10.1523/JNEUROSCI.3258-13.2013

Licastro, E., Pignataro, G., Iliff, J. J., Xiang, Y., Lo, E. H., Hayakawa, K., et al. (2024). Glymphatic and lymphatic communication with systemic responses during physiological and pathological conditions in the central nervous system. *Commun Biol* 7, 229. doi: 10.1038/s42003-024-05911-5

Lundgaard, I., Lu, M. L., Yang, E., Peng, W., Mestre, H., Hitomi, E., et al. (2017). Glymphatic clearance controls state-dependent changes in brain lactate concentration. *J Cereb Blood Flow Metab* 37, 2112–2124. doi: 10.1177/0271678X16661202

Mestre, H., Kostrikov, S., Mehta, R. I., and Nedergaard, M. (2017). Perivascular spaces, glymphatic dysfunction, and small vessel disease. *Clinical Science* 131, 2257–2274. doi: 10.1042/CS20160381

Natale, G., Limanaqi, F., Busceti, C. L., Mastroiacovo, F., Nicoletti, F., Puglisi-Allegra, S., et al. (2021). Glymphatic System as a Gateway to Connect Neurodegeneration From Periphery to CNS. *Front Neurosci* 15, 639140. doi: 10.3389/fnins.2021.639140

Ozturk, B., Koundal, S., Al Bizri, E., Chen, X., Gursky, Z., Dai, F., et al. (n.d.). Continuous positive airway pressure increases CSF flow and glymphatic transport. *JCI Insight* 8, e170270. doi: 10.1172/jci.insight.170270

Reddy, O. C., and van der Werf, Y. D. (2020). The Sleeping Brain: Harnessing the Power of the Glymphatic System through Lifestyle Choices. *Brain Sciences* 10, 868. doi: 10.3390/brainsci10110868

Sepehrband, F., Barisano, G., Sheikh-Bahaei, N., Choupan, J., Cabeen, R. P., Lynch, K. M., et al. (2021). Volumetric distribution of perivascular space in relation to mild cognitive impairment. *Neurobiology of Aging* 99, 28–43. doi: 10.1016/j.neurobiolaging.2020.12.010

Silva, I., Silva, J., Ferreira, R., and Trigo, D. (2021). Glymphatic system, AQP4, and their implications in Alzheimer's disease. *Neurol Res Pract* 3, 5. doi: 10.1186/s42466-021-00102-7

Syková, E., and Nicholson, C. (2008). Diffusion in Brain Extracellular Space. *Physiol Rev* 88, 1277–1340. doi: 10.1152/physrev.00027.2007

Szlufik, S., Kopeć, K., Szleszkowski, S., and Koziorowski, D. (2024). Glymphatic System Pathology and Neuroinflammation as Two Risk Factors of Neurodegeneration. *Cells* 13, 286. doi: 10.3390/cells13030286

Taoka, T., Masutani, Y., Kawai, H., Nakane, T., Matsuoka, K., Yasuno, F., et al. (2017). Evaluation of glymphatic system activity with the diffusion MR technique: diffusion tensor image analysis along the perivascular space (DTI-ALPS) in Alzheimer's disease cases. *Jpn J Radiol* 35, 172–178. doi: 10.1007/s11604-017-0617-z

Tian, Y., Zhao, M., Chen, Y., Yang, M., and Wang, Y. (2022). The Underlying Role of the Glymphatic System and Meningeal Lymphatic Vessels in Cerebral Small Vessel Disease. *Biomolecules* 12, 748. doi: 10.3390/biom12060748

Wardlaw, J. M., Benveniste, H., Nedergaard, M., Zlokovic, B. V., Mestre, H., Lee, H., et al. (2020). Perivascular spaces in the brain: anatomy, physiology and pathology. *Nat Rev Neurol* 16, 137–153. doi: 10.1038/s41582-020-0312-z

Xie, L., Kang, H., Xu, Q., Chen, M. J., Liao, Y., Thiyagarajan, M., et al. (2013). Sleep Drives Metabolite Clearance from the Adult Brain. *Science* 342, 373–377. doi: 10.1126/science.1241224

Supplementary Table 1. PRISMA 2020 Checklist

Section and Topic	Item #	Checklist item	Location where item is reported
**TITLE**			
Title	1	Identify the report as a systematic review.	p.01, Title
**ABSTRACT**			
Abstract	2	See the PRISMA 2020 for Abstracts checklist.	p.01, Abstract
**INTRODUCTION**			
Rationale	3	Describe the rationale for the review in the context of existing knowledge.	p.02, S1
Objectives	4	Provide an explicit statement of the objective(s) or question(s) the review addresses.	p.02, S1, 2.1
**METHODS**			
Eligibility criteria	5	Specify the inclusion and exclusion criteria for the review and how studies were grouped for the syntheses.	p.02, S2.1, 2.2
Information sources	6	Specify all databases, registers, websites, organisations, reference lists and other sources searched or consulted to identify studies. Specify the date when each source was last searched or consulted.	p.02, S2.1
Search strategy	7	Present the full search strategies for all databases, registers and websites, including any filters and limits used.	p.02, S2.1
Selection process	8	Specify the methods used to decide whether a study met the inclusion criteria of the review, including how many reviewers screened each record and each report retrieved, whether they worked independently, and if applicable, details of automation tools used in the process.	p.02-03, S2.3
Data collection process	9	Specify the methods used to collect data from reports, including how many reviewers collected data from each report, whether they worked independently, any processes for obtaining or confirming data from study investigators, and if applicable, details of automation tools used in the process.	p.03, S2.3
Data items	10a	List and define all outcomes for which data were sought. Specify whether all results that were compatible with each outcome domain in each study were sought (e.g. for all measures, time points, analyses), and if not, the methods used to decide which results to collect.	p.02, S2.1<br>p.03, S2.3
	10b	List and define all other variables for which data were sought (e.g. participant and intervention characteristics, funding sources). Describe any assumptions made about any missing or unclear information.	p.03, S2.3
Study risk of bias assessment	11	Specify the methods used to assess risk of bias in the included studies, including details of the tool(s) used, how many reviewers assessed each study and whether they worked independently, and if applicable, details of automation tools used in the process.	p.03-04, S2.4; Supp. Tab. 2-7
Effect measures	12	Specify for each outcome the effect measure(s) (e.g. risk ratio, mean difference) used in the synthesis or presentation of results.	p.04, S2.5; Tab. 2
Synthesis methods	13a	Describe the processes used to decide which studies were eligible for each synthesis (e.g. tabulating the study intervention characteristics and comparing against the planned groups for each synthesis (item #5)).	p.04, S2.5
	13b	Describe any methods required to prepare the data for presentation or synthesis, such as handling of missing summary statistics, or data conversions.	p.04, S2.5
	13c	Describe any methods used to tabulate or visually display results of individual studies and syntheses.	p.04, S2.5; Fig 2-4
	13d	Describe any methods used to synthesize results and provide a rationale for the choice(s). If meta-analysis was performed, describe the model(s), method(s) to identify the presence and extent of statistical heterogeneity, and software package(s) used.	p.04, S2.5
	13e	Describe any methods used to explore possible causes of heterogeneity among study results (e.g. subgroup analysis, meta-regression).	p.04, S2.5
	13f	Describe any sensitivity analyses conducted to assess robustness of the synthesized results.	p.04, S2.5; Tab. 2
Reporting bias assessment	14	Describe any methods used to assess risk of bias due to missing results in a synthesis (arising from reporting biases).	p.04, S2.5;<br>Supp. Fig 3-5
Certainty assessment	15	Describe any methods used to assess ainty (or confidence) in the body of evidence for an outcome.	p.03-04, S2.4;<br>Supp. Tab. 2-7
**RESULTS** cert			
Study selection	16a	Describe the results of the search and selection process, from the number of records identified in the search to the number of studies included in the review, ideally using a flow diagram.	p.04, S3.1; Fig 1
	16b	Cite studies that might appear to meet the inclusion criteria, but which were excluded, and explain why they were excluded.	p.04, S3.1
Study characteristics	17	Cite each included study and present its characteristics.	p.04, S3.2; Tab. 1

PRISMA 2020 Checklist

Section and Topic	Item #	Checklist item	Location where item is reported
Risk of bias in studies	18	Present assessments of risk of bias for each included study.	p.04, S3.1; Supp. Tab. 2-7
Results of individual studies	19	For all outcomes, present, for each study: (a) summary statistics for each group (where appropriate) and (b) an effect estimate and its precision (e.g. confidence/credible interval), ideally using structured tables or plots.	p.04-17, S3.2-3.6; Fig 2-4; Tab. 1,2
Results of syntheses	20a	For each synthesis, briefly summarise the characteristics and risk of bias among contributing studies.	p.04, S3.2; p.04-17, S3.3-3.6
	20b	Present results of all statistical syntheses conducted. If meta-analysis was done, present for each the summary estimate and its precision (e.g. confidence/credible interval) and measures of statistical heterogeneity. If comparing groups, describe the direction of the effect.	p.04-15, S3.3-3.4; Tab. 2; Fig 2-4
	20c	Present results of all investigations of possible causes of heterogeneity among study results.	p.13-16, S3.3-3.5
	20d	Present results of all sensitivity analyses conducted to assess the robustness of the synthesized results.	p.13-15, S3.3-3.4; Tab. 2; Supp. Fig 3-5
Reporting biases	21	Present assessments of risk of bias due to missing results (arising from reporting biases) for each synthesis assessed.	p.13-15, S3.3-3.4; Supp. Fig 3-5
Certainty of evidence	22	Present assessments of certainty (or confidence) in the body of evidence for each outcome assessed.	p.21, S4.4; Supp. Tab. 2-7
**DISCUSSION**			
Discussion	23a	Provide a general interpretation of the results in the context of other evidence.	p.17-20, S4.1-4.2;
	23b	Discuss any limitations of the evidence included in the review.	p.21, S4.4;
	23c	Discuss any limitations of the review processes used.	p.21, S4.4;
	23d	Discuss implications of the results for practice, policy, and future research.	p.19-21, S4.4, S5;
**OTHER INFORMATION**			
Registration and protocol	24a	Provide registration information for the review, including register name and registration number, or state that the review was not registered.	p.02, S2;
	24b	Indicate where the review protocol can be accessed, or state that a protocol was not prepared.	p.02, S2;
	24c	Describe and explain any amendments to information provided at registration or in the protocol.	Not reported
Support	25	Describe sources of financial or non-financial support for the review, and the role of the funders or sponsors in the review.	p.21, Funding;
Competing interests	26	Declare any competing interests of review authors.	p.21, CoI;
Availability of data, code and other materials	27	Report which of the following are publicly available and where they can be found: template data collection forms; data extracted from included studies; data used for all analyses; analytic code; any other materials used in the review.	p.21, DVS; p.22, Supp. material

From: Page MJ, McKenzie JE, Bossuyt PM, Boutron I, Hoffmann TC, Mulrow CD, et al. The PRISMA 2020 statement: an updated guideline for reporting systematic reviews. BMJ 2021;372:n71. doi: 10.1136/bmj.n71. This work is licensed under CC BY 4.0. To view a copy of this license, visit https://creativecommons.org/licenses/by/4.0/

Supplementary Table 2. Detailed quality assessment of included studies based on the Newcastle-Ottawa Scale (NOS)

Ref.	study type	Selection				Comparability	Exposure			Total Score
		Item 1	Item 2	Item 3	Item 4	Item 5	Item 6	Item 7	Item 8	
(Taoka et al., 2017)	1	*	/	/	*	/	*	*	/	4
(Steward et al., 2021)	1	*	/	/	*	**	*	*	/	6
(Ota et al., 2022)	1	*	/	/	*	*	*	*	/	5
(Hsu et al., 2023)	1	*	/	/	*	**	*	*	/	6
(Liang et al., 2023)	1	*	/	/	*	*	*	*	/	5
(Chang et al., 2023)	1	*	/	*	*	**	*	*	/	7
(Saito, Kamagata, Andica, Taoka, et al., 2023)	1	*	/	/	*	**	*	*	/	6
(Huang et al., 2024)	2	*	*	*	*	**	*	*	/	8
(X. Zhang et al., 2024)	1	*	/	*	*	**	*	*	/	7
(Y. Li et al., 2024)	2	*	*	*	*	**	*	*	/	8
(Hong et al., 2024)	1	*	/	/	*	**	*	*	/	6
(Sacchi et al., 2024)	1	*	/	/	*	**	*	*	/	6
(Shang et al., 2024)	1	*	/	*	*	**	*	*	/	7
(Kim et al., 2024)	1	*	/	/	*	**	*	*	/	6
(Okazawa et al., 2024)	1	*	/	/	*	*	*	*	/	5
(Xia et al., 2025)	1	*	/	/	*	**	*	*	/	6
(Y. Zhang et al., 2025)	1	*	/	/	*	**	*	*	/	6
(Schirge et al., 2025)	1	*	/	/	*	**	*	*	/	6
(S. Li et al., 2025)	1	*	/	/	*	**	*	*	/	6
(Z. Chen et al., 2025)	1	*	/	/	*	*	*	*	/	5
(You, Kim, Hwang, et al., 2025)	1	*	/	/	*	**	*	*	/	6
(Luo et al., 2025)	1	*	/	/	*	**	*	*	/	6
(Q. Zhang et al., 2025)	1	*	/	*	*	**	*	*	/	7
(Wang et al., 2025)	1	*	/	/	*	**	*	*	/	6
(Firbank et al., 2025)	1	*	/	/	*	**	*	*	/	6
(Jiao et al., 2025)	1	*	/	/	*	**	*	*	/	6
(Guo et al., 2025)	2	*	*	*	*	**	*	*	/	8
(Chang et al., 2025)	1	*	/	*	*	**	*	*	/	7
(Agah et al., 2025)	2	*	*	*	*	**	*	*	*	9
(Jungwon et al., 2025)	2	/	*	*	/	**	*	*	/	6
(Wu et al., 2025)	2	*	*	*	/	**	*	/	/	6
(You, Kim, Kim, et al., 2025)	1	*	/	/	*	**	*	*	/	6
(Yu et al., 2025)	1	*	/	/	*	**	*	*	/	6
(Lin et al., 2025)	1	*	/	*	*	**	*	*	/	6
(Bao et al., 2025)	2	*	*	*	*	**	*	*	*	9
(F. Chen et al., 2025)	1	*	/	*	*	**	*	*	/	6

Study Type: 1 = Case-Control Study; 2 = Cohort Study; Scoring: *: Item received one star (indicating low risk of bias); /: Item received no star.
For Case-Control Studies (Type 1): Selection: Item 1: Is the case definition adequate? Item 2: Representativeness of the cases; Item 3: Selection of controls; Item 4: Definition of controls. Comparability: Item 5: Comparability of cases and controls on the basis of the design or analysis (Maximum 2 stars). Exposure: Item 6: Ascertainment of exposure; Item 7: Same method of ascertainment for cases and controls; Item 8: Non-Response rate.
For Cohort Studies (Type 2): Selection: Item 1: Representativeness of the exposed cohort; Item 2: Selection of the non-exposed cohort; Item 3: Ascertainment of exposure; Item 4: Demonstration that outcome of interest was not present at start of study. Comparability: Item 5: Comparability of cohorts on the basis of the design or analysis (Maximum 2 stars). Outcome: Item 6: Assessment of outcome; Item 7: Was follow-up long enough for outcomes to occur? Item 8: Adequacy of follow-up of cohorts.

Supplementary Table 3. Domain 1: Study eligibility criteria

Signalling question	Rating	Reasoning
1.1 Did the review adhere to pre-defined objectives and eligibility criteria?	Yes	The authors explicitly stated the objectives in the Abstract and Introduction: to synthesize evidence on DTI-ALPS in AD, clarify its role in pathophysiology, examine controversies, and outline future directions. The methods section details pre-defined eligibility criteria (original human studies, AD patients vs. controls, use of DTI-ALPS, and specific analyses).
1.2 Were the eligibility criteria appropriate for the review question?	Yes	The criteria (population: AD/MCI/HC; index test: DTI-ALPS; outcomes: association with A/T/N biomarkers and cognition) are directly aligned with the review's objective of evaluating DTI-ALPS as a biomarker within the AD pathological framework.
1.3 Were eligibility criteria unambiguous?	Yes	The inclusion and exclusion criteria are clearly listed and specific. They define the study designs (original research), participants (human, with specific diagnostic groups), intervention/index test (DTI-ALPS calculation), and required analyses (group differences or correlations).
1.4 Were all restrictions in eligibility criteria based on study characteristics appropriate?	Yes	Restrictions excluding animal studies, non-English articles, preprints, conference proceedings, and dissertations are standard and appropriate for a systematic review aiming to synthesize published, peer-reviewed human clinical research.
1.5 Were any restrictions in eligibility criteria based on sources of information appropriate?	Probably Yes	The exclusion of non-English articles is a common restriction, though it may introduce language bias. The authors did not apply other restrictions on sources, and the justification, while not explicitly stated, is pragmatically accepted in many systematic reviews.
Concerns regarding specification of study eligibility criteria	Low	
Rationale for concern	All signalling questions were answered "Yes" or “Probably Yes”. The review question, objectives, and detailed eligibility criteria were clearly pre-specified, which minimizes concerns about the specification of study eligibility criteria.	

Supplementary Table 4. Domain 2: Identification and selection of studies

Signalling question	Rating	Reasoning
2.1 Did the search include an appropriate range of databases/ electronic sources for published and unpublished reports?	Yes	The authors searched three major bibliographic databases: Web of Science, PubMed, and ScienceDirect. This represents a good range of sources for identifying published literature in the biomedical and life sciences.
2.2 Were methods additional to database searching used to identify relevant reports?	No	The manuscript does not mention supplementary search methods such as checking reference lists of included studies or relevant reviews, contacting experts, or searching trial registries. This could be a potential limitation.
2.3 Were the terms and structure of the search strategy likely to retrieve as many eligible studies as possible?	Yes	The search strategy employed comprehensive keywords capturing all known acronyms and full names for both the index test ("DTI-ALPS", "diffusion tensor imaging along the perivascular space", "ALPS") and the disease ("Alzheimer's disease", "AD", "Alzheimer"). The use of Boolean "OR" within concepts and "AND" between concepts is a sound and sensitive approach to maximize retrieval. This strategy is highly likely to have captured the vast majority of relevant published records on this specific topic.
2.4 Were restrictions based on date, publication format, or language appropriate?	Yes	The search was restricted from January 1, 2017 (the year DTI-ALPS was introduced) to the search date (September 18, 2025), which is logical. The exclusion of preprints and conference proceedings is a justifiable restriction on publication format.
2.5 Were efforts made to minimise errors in selection of studies?	Yes	The process was conducted independently by two researchers with cross-verification. Disagreements were resolved through discussion or by a third researcher, which is a robust method to minimize selection errors.
Concerns regarding methods used to identify and/or select studies	Low	
Rationale for concern	Despite the lack of reported supplementary search methods, the use of major databases, a clear time frame, and a rigorous, independent study selection process with dual review suggests a low risk of missing relevant studies or introducing selection bias.	

Supplementary Table 5. Domain 3: Data collection and study appraisal

Signalling question	Rating	Reasoning
3.1 Were efforts made to minimise error in data collection?	Yes	Two researchers independently extracted data using a pre-designed standardized form. Discrepancies were resolved through discussion or arbitration by a third researcher, effectively minimizing errors.
3.2 Were sufficient study characteristics available for both review authors and readers to be able to interpret the results?	Yes	The authors state that Table 1 provides a standardized summary of data from included studies. Key elements like first author, sample size, MRI modality, and main results were extracted, allowing for interpretation.
3.3 Were all relevant study results collected for use in the synthesis?	Yes	The data extraction form was designed to capture all relevant results, including those related to group differences, correlations with A/T/N biomarkers, and cognitive scores, which formed the basis of the narrative synthesis.
3.4 Was risk of bias (or methodological quality) formally assessed using appropriate criteria?	Yes	The methodological quality and risk of bias of the included studies were independently assessed by two researchers using the Newcastle-Ottawa Scale (NOS). Discrepancies were resolved through consensus.
3.5 Were efforts made to minimise error in risk of bias assessment?	Yes	Two investigators independently assessed study quality.
Concerns regarding methods used to collect data and appraise studies	Low	
Rationale for concern	All signaling questions were rated as “Yes” and so no potential areas of bias were identified. The review processes of data collection and study appraisal are therefore unlikely to have introduced bias into this review.	

Supplementary Table 6. Domain 4: Synthesis and findings

Signalling question	Rating	Reasoning
4.1 Did the synthesis include all studies that it should?	Yes	The narrative synthesis is based on all 36 included studies, as referenced throughout the results and discussion sections. The flow diagram (Fig 1) accounts for all identified and included records.
4.2 Were all predefined analyses followed or departures explained?	Yes	The synthesis followed the structure outlined in the methods and results sections (e.g., early validation, multimodal associations, dynamic role, methodological advancements). No major departures from the planned synthesis were noted.
4.3 Was the synthesis appropriate given the nature and similarity in the research questions, study designs and outcomes across included studies?	Yes	Random-effects meta-analyses were conducted when studies employed comparable methodologies and reported effect sizes (e.g., correlations with Aβ/tau, cognitive scores, and group differences). A narrative synthesis was performed only for studies not suitable for quantitative synthesis.
4.4 Was between-studies variation (heterogeneity) minimal or addressed in the synthesis?	Yes	The authors explicitly acknowledged heterogeneity as the reason for choosing a narrative synthesis. They thoroughly discussed potential sources of heterogeneity throughout the discussion to explain discrepant findings.
4.5 Was robustness of the finding(s) assessed e.g. through funnel plot or sensitivity analyses?	Yes	The robustness of findings was rigorously assessed. Publication bias was evaluated using funnel plots, Egger's linear regression test, and the Trim and Fill method. Additionally, meta-regression analyses were conducted to explore sources of heterogeneity (e.g., age, ROI placement).
4.6 Were biases in primary studies minimal or addressed in the synthesis?	Yes	The authors critically appraised and discussed methodological limitations of the primary studies (e.g., lack of standardization, confounding by white matter microstructure) as a central theme of the review, effectively addressing how these biases impact the overall evidence.
Concerns regarding the synthesis and findings	Low	
Rationale for concern	The synthesis method was appropriate for the available data. The authors provided a thorough and critical discussion of the evidence, explicitly addressing heterogeneity and biases in the primary literature. The conclusions are supported by the presented synthesis.	

Supplementary Table 7. Judging risk of bias

**Domain**	**Concern**	**Rationale for concern**
1. Concerns regarding specification of study eligibility criteria	Low	All signalling questions were answered "Yes" or “Probably Yes”. The review question, objectives, and detailed eligibility criteria were clearly pre-specified, which minimizes concerns about the specification of study eligibility criteria.
2. Concerns regarding methods used to identify and/or select studies	Low	Despite the lack of reported supplementary search methods, the use of major databases, a clear time frame, and a rigorous, independent study selection process with dual review suggests a low risk of missing relevant studies or introducing selection bias.
3. Concerns regarding used to collect data and appraise studies	Low	All signaling questions were rated as “Yes” and so no potential areas of bias were identified. The review processes of data collection and study appraisal are therefore unlikely to have introduced bias into this review.
4. Concerns regarding the synthesis and findings	Low	The synthesis method included both quantitative meta-analysis and narrative synthesis, which was appropriate for the available data. The authors assessed heterogeneity using I^2 and meta-regression, and evaluated publication bias using statistical tests (e.g., Trim and Fill), ensuring the robustness of the conclusions.

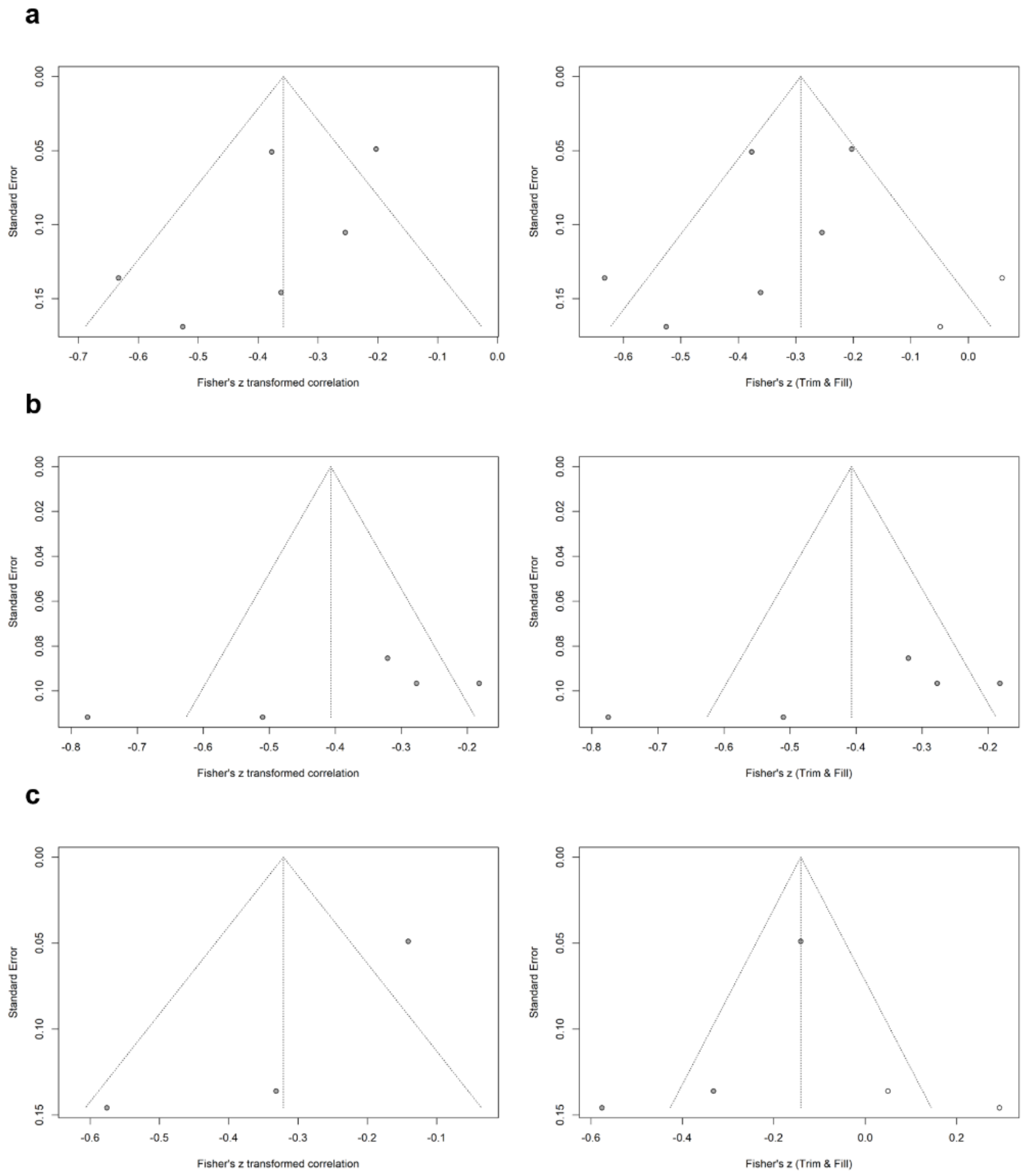

Supplementary Figure 3. Funnel and Trim Fill plots illustrating the associations between the DTI-ALPS index and core AD protein biomarkers. a Studies using partial correlation analysis to assess the association between the DTI-ALPS index and Aβ PET deposition. b Studies using correlation analysis to assess the association between the DTI-ALPS index and Aβ PET deposition. c Studies using partial correlation analysis to assess the association between the DTI-ALPS index and tau PET deposition

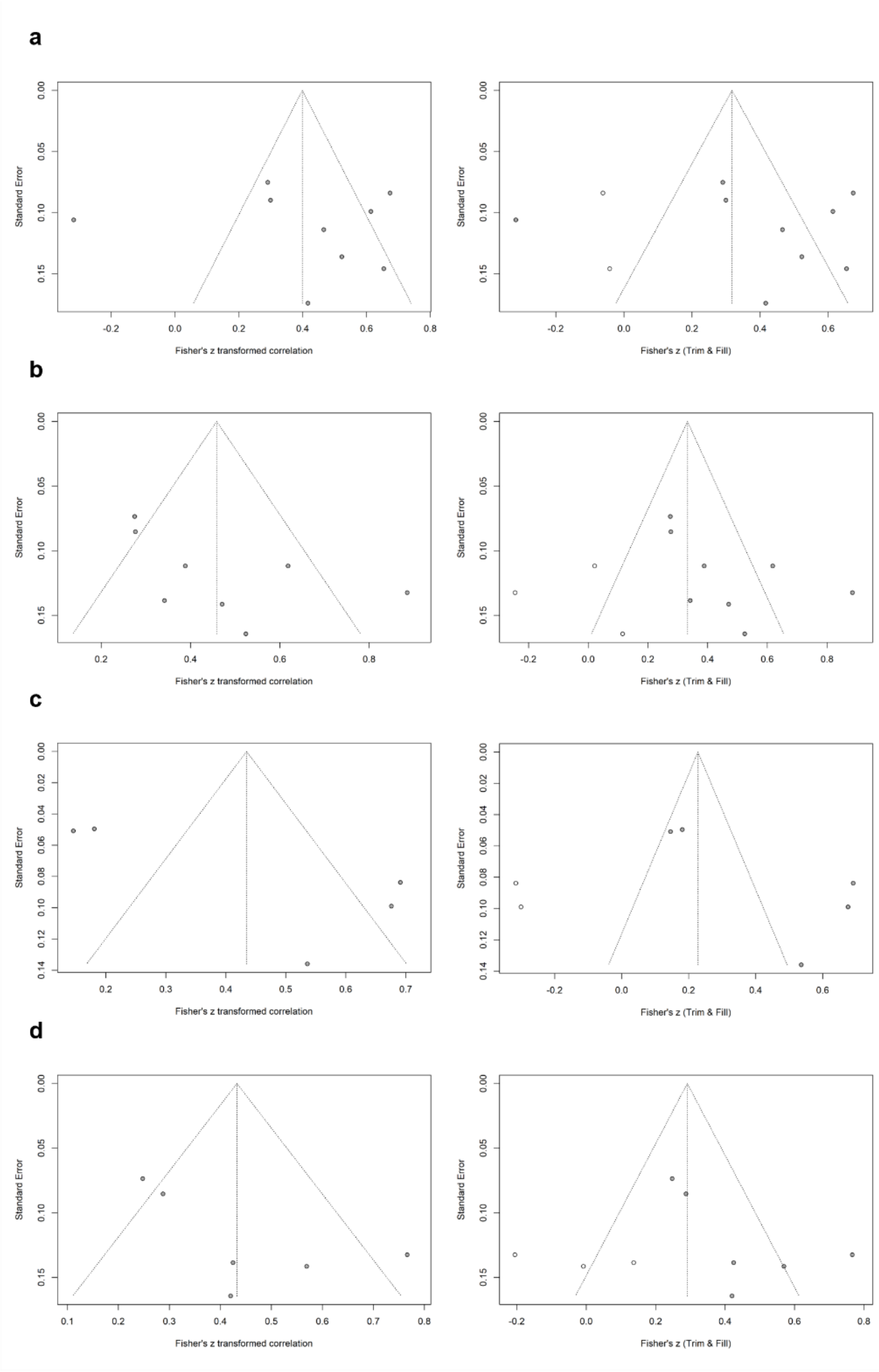

Supplementary Figure 4. Funnel and Trim Fill plots illustrating the associations between the DTI-ALPS index and cognitive function. a Studies using partial correlation analysis to assess the association between the DTI-ALPS index and MMSE scores. b Studies using correlation analysis to assess the association between the DTI-ALPS index and MMSE scores. c Studies using partial correlation analysis to assess the association between the DTI-ALPS index and MoCA scores. d Studies using correlation analysis to assess the association between the DTI-ALPS index and MoCA scores

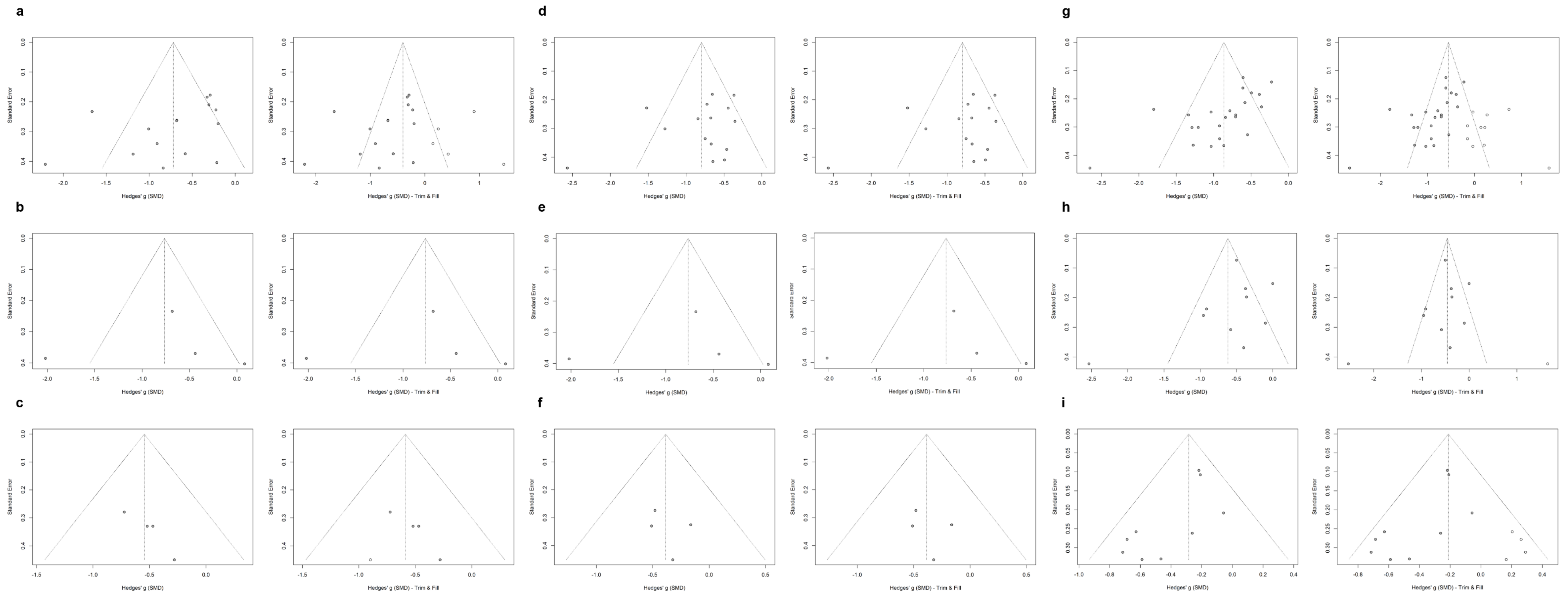

Supplementary Figure 5. Funnel and Trim Fill plots illustrating differences in the DTI-ALPS index across diagnostic subgroups. a–c Comparisons of the left DTI-ALPS index between AD and CN (a), AD and MCI (b), and MCI and CN (c) groups. d–f Comparisons of the right DTI-ALPS index between AD and CN (d), AD and MCI (e), and MCI and CN (f) groups. g–i Comparisons of the mean DTI-ALPS index between AD and CN (g), AD and MCI (h), and MCI and CN (i) groups
